# Tau PET positivity predicts clinically relevant cognitive decline driven by Alzheimer’s disease compared to comorbid cases; proof of concept in the ADNI study

**DOI:** 10.1038/s41380-024-02672-9

**Published:** 2024-08-23

**Authors:** Konstantinos Ioannou, Marco Bucci, Antonios Tzortzakakis, Irina Savitcheva, Agneta Nordberg, Konstantinos Chiotis, for the Alzheimer’s Disease Neuroimaging Initiative

**Affiliations:** 1 Division of Clinical Geriatrics, Center for Alzheimer Research, Department of Neurobiology, Care Sciences and Society, Karolinska Institutet, Stockholm, Sweden; 2 Theme Inflammation and Aging, Karolinska University Hospital, Stockholm, Sweden; 3 Division of Radiology, Department for Clinical Science, Intervention and Technology (CLINTEC), Karolinska Institutet, Stockholm, Sweden; 4 Medical Radiation Physics and Nuclear Medicine, Section for Nuclear Medicine, Karolinska University Hospital, Stockholm, Sweden; 5 Department of Neurology, Karolinska University Hospital, Stockholm, Sweden

**Keywords:** Prognostic markers, Neuroscience

## Abstract

β-amyloid (Aβ) pathology is not always coupled with Alzheimer’s disease (AD) relevant cognitive decline. We assessed the accuracy of tau PET to identify Aβ(+) individuals who show prospective disease progression. 396 cognitively unimpaired and impaired individuals with baseline Aβ and tau PET and a follow-up of ≥ 2 years were selected from the Alzheimer’s Disease Neuroimaging Initiative dataset. The participants were dichotomously grouped based on either clinical conversion (i.e., change of diagnosis) or cognitive deterioration (fast (FDs) vs. slow decliners (SDs)) using data-driven clustering of the individual annual rates of cognitive decline. To assess cognitive decline in individuals with isolated Aβ(+) or absence of both Aβ and tau (T) pathologies, we investigated the prevalence of non-AD comorbidities and FDG PET hypometabolism patterns suggestive of AD. Baseline tau PET uptake was higher in Aβ(+)FDs than in Aβ(-)FD/SDs and Aβ(+)SDs, independently of baseline cognitive status. Baseline tau PET uptake identified MCI Aβ(+) Converters and Aβ(+)FDs with an area under the curve of 0.85 and 0.87 (composite temporal region of interest) respectively, and was linearly related to the annual rate of cognitive decline in Aβ(+) individuals. The T(+) individuals constituted largely a subgroup of those being Aβ(+) and those clustered as FDs. The most common biomarker profiles in FDs (*n* = 70) were Aβ(+)T(+) (*n* = 34, 49%) and Aβ(+)T(-) (*n* = 19, 27%). Baseline Aβ load was higher in Aβ(+)T(+)FDs (M = 83.03 ± 31.42CL) than in Aβ(+)T(-)FDs (M = 63.67 ± 26.75CL) (*p*-value = 0.038). Depression diagnosis was more prevalent in Aβ(+)T(-)FDs compared to Aβ(+)T(+)FDs (47% vs. 15%, *p*-value = 0.021), as were FDG PET hypometabolism pattern not suggestive of AD (86% vs. 50%, *p*-value = 0.039). Our findings suggest that high tau PET uptake is coupled with both Aβ pathology and accelerated cognitive decline. In cases of isolated Aβ(+), cognitive decline may be associated with changes within the AD spectrum in a multi-morbidity context, i.e., mixed AD.

## Introduction

β-amyloid (Aβ) positron emission tomography (PET) can accurately detect Αβ plaques in vivo [1] and has improved diagnostic confidence, refining Alzheimer’s disease (AD) patient management [2, 3]. However, the clinical utility of Αβ PET is mainly defined by its high negative predictive value (80–100%) [4, 5] i.e., a negative result can rule out AD and is significantly more meaningful than a positive one in clinical decision-making [6]. The presence of Αβ pathology is a common finding in the elderly, and it is not deterministic for prospective cognitive deterioration [7–9]. The relatively low positive predictive value of Aβ biomarkers [4, 10], in combination with the presence of Aβ pathology in other neurodegenerative disorders [11–13], challenges the interpretation of the results of ongoing clinical trials, which have focused on the recruitment of individuals in the AD spectrum based solely on Aβ positivity (Αβ(+)), even in the absence of cognitive symptoms. There is thus a warranted need for integrating novel biomarkers with higher prognostic accuracy into clinical practice for more accurate identification of clinically relevant AD pathology.

Earlier in vivo [14, 15] and autopsy studies [16] have shown a strong relationship of tau, rather than Aβ, pathology with AD neurodegeneration and cognitive performance. Further PET imaging studies have highlighted a temporal offset in this correlation, with tau deposition preceding local neuronal loss [8, 15] and being predictive of future cognitive decline [17–19]. In line with this temporal sequence, there is compelling evidence that T(+) individuals could be largely a subgroup of those being Aβ(+) [20]. The interplay between Aβ and tau pathology, as imaged with PET, has also been assessed [21–23], and the concurrent presence of both pathologies has been associated with a higher risk for fast decline relative to the presence of only Aβ [17, 20]. Nonetheless, the widespread adoption of the full characterization of an individual based on both Aβ and tau PET biomarkers appears an unrealistic clinical policy, given the associated costs and the required healthcare resources.

Despite highlighting the association between tau load and prospective cognitive decline [15, 17, 24], studies employing longitudinal cognition as a continuous variable do not allow assessment of the prognostic accuracy of the biomarker and, therefore, its ability to predict a cognitive trajectory at the individual level. Furthermore, both the use of group-level association analyses and the small sample sizes [15, 18, 25] with clinically relevant follow-up intervals do not allow elaborate assessment of the presence of other factors that could affect cognitive deterioration at an individual basis and explain concordant and discordant results between Aβ and tau biomarkers. Personalized interpretation of a biomarker result holds important clinical implications. Addressing this inquiry constitutes a crucial, yet pending, step for the establishment of tau biomarkers. To further expand the available evidence on the prognostic accuracy of tau biomarkers at an individual level, one could take advantage of the bimodal distribution of cognitive trajectories observed in both aging and clinical studies, and evaluate the accuracy of tau biomarkers to discriminate individuals declining from those remaining stable [26–28].

We aimed to assess the clinical utility of tau PET imaging as a standalone predictor of AD-relevant cognitive decline by testing its ability to detect the subgroup of Aβ(+) individuals who experience prospective disease progression in Alzheimer’s Disease Neuroimaging Initiative (ADNI) participants. Our study encompasses two distinct objectives: 1) clustering the individuals based on disease progression considering both change in clinical diagnosis and the rate of cognitive decline, and evaluating the accuracy of baseline tau PET for discriminating individuals with an accelerated profile of prospective cognitive decline; and 2) understanding the determinants of cognitive decline in individuals with discordant Aβ and tau biomarker status (i.e., isolated Aβ(+)) by investigating the prevalence of non-AD comorbidities and 18F-fluorodeoxyglucose (FDG) PET hypometabolism patterns suggestive of AD. We hypothesized that if tau PET is positive only in Aβ(+) individuals with AD-relevant cognitive decline, then in cases of isolated Aβ(+), cognitive decline could be associated with changes within the AD spectrum (i.e., the presence of Aβ pathology) in a mixed pathology context. The alternative hypothesis posits that if isolated Aβ(+) is solely associated with AD-relevant cognitive decline, then tau biomarkers may lack the necessary sensitivity, and would therefore have limited clinical utility.

## Methods

### Individuals

The data used for our analysis were obtained from the ADNI repository (https://adni.loni.usc.edu) on 21 January 2022 (Supplementary Table 1). ADNI is a multicentered study launched in 2003 and run as a public-private partnership led by Principal Investigator Michael W. Weiner. The primary aim of ADNI is to test whether imaging techniques, biological markers and clinical and neuropsychological assessment can be combined to measure the progression of mild cognitive impairment (MCI) and early AD.

**Fig. 1:**
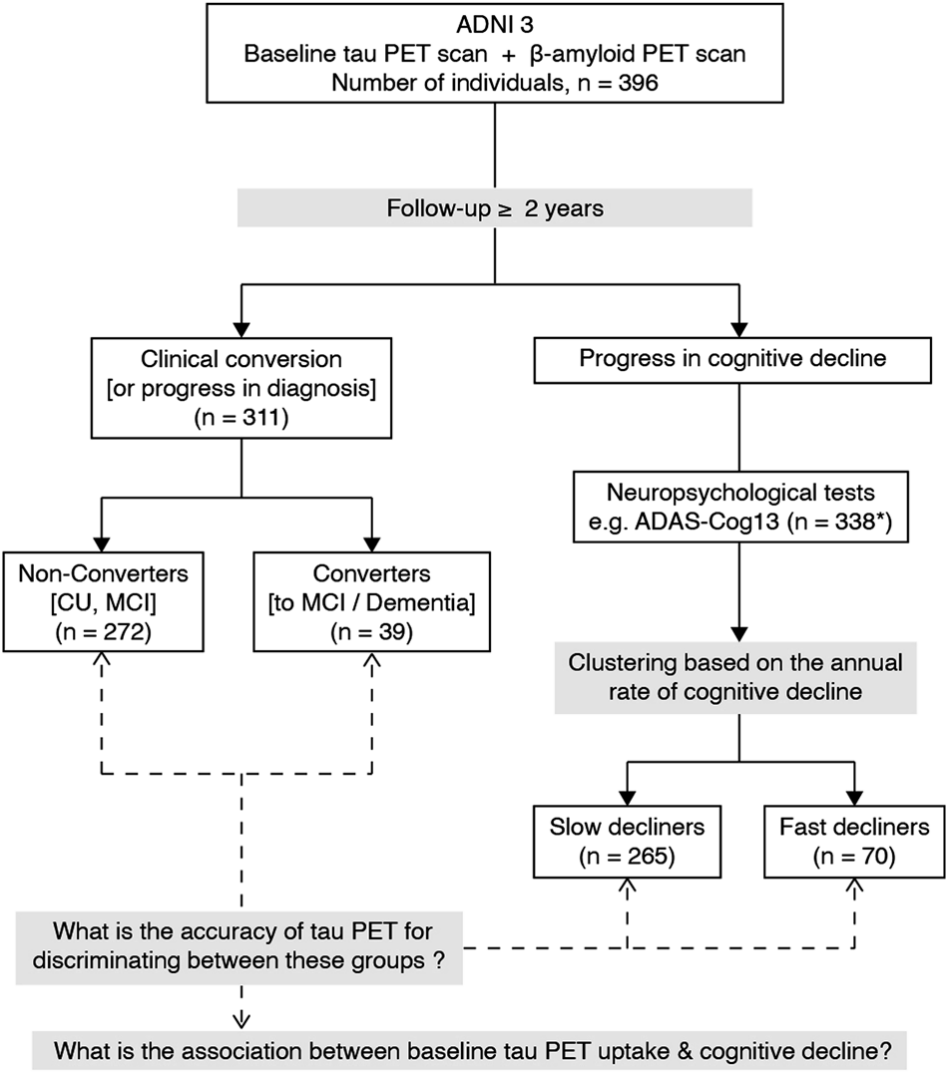
A flow chart of the analysis pathway. Both clinical conversion and progress in cognitive decline were studied (* number of individuals before the exclusion of three outliers/influential points). ADAS-Cog13 13-item version of the Alzheimer’s Disease Assessment Scale-Cognitive Subscale; ADNI Alzheimer’s Disease Neuroimaging Initiative, CU cognitively unimpaired, MCI mild cognitive impairment, PET positron emission tomography.

We identified all participants from the latest phase of ADNI (ADNI3) who had undergone at least one tau and one Aβ PET scan within six months and had available longitudinal clinical and cognitive follow-up over a minimum of two years (Fig. 1, Table 1). Based on their clinical diagnosis at the time of the baseline tau PET scan (i.e., a priori baseline time point), the individuals were grouped into cognitively unimpaired (CU) or impaired (CI) (i.e., individuals diagnosed with MCI or dementia).

**Table 1:** Clinical characteristics by baseline clinical diagnosis and β-amyloid PET status.

β-amyloid PET	CU		MCI		Dementia		*p*-values^c^
	Aβ(-) *(N* = *170)*^a^	Aβ(+) *(N* = *81)*^a^	Aβ(-) *(N* = *61)*^a^	Aβ(+) *(N* = *57)*^a^	Aβ(-)^b^*(N* = *4)*^a^	Aβ(+) *(N* = *23)*^a^	
**Baseline**							
**Age, y**	71.75 (6.57)	75.76 (7.21)	74.30 (8.88)	74.25 (6.75)	70.26 (8.08)	76.42 (9.17)	**<0.001** ***
**Sex**							**0.003** **
Male	64 (38%)	32 (40%)	39 (64%)	34 (60%)	2 (50%)	13 (57%)	–
Female	106 (62%)	49 (60%)	22 (36%)	23 (40%)	2 (50%)	10 (43%)	–
**Education, y**	16.95 (2.25)	16.44 (2.31)	16.10 (2.76)	16.21 (2.56)	15 (3.46)	15.65 (2.71)	**0.046** *
**MMSE (379 individuals)**							
Baseline	29.22 (0.96)	28.86 (1.37)	28.62 (1.54)	27.33 (2.26)	24.75 (1.26)	22.50 (3.42)	**<0.001** ***
T. Interval, mon	35.95 (12.73)	35.41 (11.12)	37.38 (11.30)	36.51 (9.85)	25.94 (1.13)	29.73 (7.24)	0.15
Missing data	9 (5%)	4 (5%)	3 (5%)	0 (0%)	0 (0%)	1 (4%)	0.4
**Longitudinal cognitive measures**							
**ADAS-Cog13 (338 individuals)**							
Baseline	7.43 (4.39)	8.38 (4.62)	11.88 (4.57)	15.88 (6.62)	29.55 (7.69)	29.33 (10.27)	**<0.001** ***
Follow-up	8.12 (5.65)	9.78 (5.49)	12.95 (6.66)	21.37 (11.14)	27.33 (10.12)	38.59 (14.88)	**<0.001** ***
Score Diff.	0.70 (3.72)	1.39 (4.55)	1.08 (4.20)	5.49 (7.67)	–2.22 (3.01)	9.26 (10.78)	**<0.001** ***
T. Interval, mon	36.22 (13.07)	35.6 (10.15)	37.77 (11.19)	37.33 (10.00)	25.64 (1.58)	29.62 (8.00)	0.13
Missing data	25 (15%)	8 (10%)	10 (16%)	9 (16%)	1 (25%)	5 (22%)	0.6
**ADNI MEM (325 individuals)**							
Baseline	1.13 (0.60)	1.03 (0.57)	0.68 (0.59)	0.19 (0.65)	–0.58 (0.70)	–0.61 (0.69)	**<0.001** ***
Follow-up	1.28 (0.86)	1.03 (0.72)	0.62 (0.86)	–0.23 (1.05)	–0.53 (0.79)	–1.23 (1.00)	**<0.001** ***
Score Diff.	0.15 (0.62)	0.01 (0.49)	–0.06 (0.47)	–0.42 (0.60)	0.05 (0.26)	–0.62 (0.73)	**<0.001** ***
T. Interval, mon	33.95 (11.72)	34.15 (9.82)	38.8 (11.89)	36.63 (9.97)	25.64 (1.58)	29.19 (8.02)	**0.039** *
Missing data	29 (17%)	9 (11%)	16 (26%)	11 (19%)	1 (25%)	5 (22%)	0.2
**PACC (326 individuals)**							
Baseline	1.01 (2.55)	–0.23 (2.89)	–2.40 (3.41)	-5.36 (4.30)	–10.89 (6.39)	–14.32 (5.78)	**<0.001** ***
Follow-up	0.75 (3.25)	–0.97 (3.83)	–2.91 (5.12)	-8.63 (6.89)	–11.92 (7.58)	-22.3 (11.60)	**<0.001** ***
Score Diff.	–0.26 (2.32)	–0.74 (2.91)	–0.51 (3.07)	–3.27 (4.08)	–1.03 (2.95)	–7.98 (7.80)	**<0.001** ***
T. Interval, mon	34.26 (11.77)	33.84 (10.35)	38.75 (11.76)	36.88 (10.31)	25.64 (1.58)	29.19 (8.02)	**0.037** *
Missing data	29 (17%)	9 (11%)	15 (25%)	11 (19%)	1 (25%)	5 (22%)	0.3
**Assessment of clinical conversion for 311 individuals (total number of individuals during clinical follow-up** = **332)**^d^							
Individuals, *N*	146	71	48	46	3	18	
**Clinical Conversion**							**<0.001** ***
Non-Converters	137 (94%)	61 (86%)	46 (96%)	28 (61%)	–	–	
Converters	9 (6%)	10 (14%)	2 (4%)	18 (39%)	–	–	
T. Interval, mon	35.67 (12.55)	34.09 (10.54)	38.71 (11.51)	36.72 (10.05)	–	–	0.2

^a^Mean (Standard deviation); *n*/*N* (%).

^b^Dementia with Aβ(-) was excluded from the calculation of *p*-values due to the small number of individuals.

^c^Kruskal-Wallis rank sum test and Fisher’s Exact Test for count data with simulated *p*-value (based on 2000 replicates) were used. The false discovery rate method was used for multiple comparisons.

^d^Because of missing data, the number of individuals at follow-up was different in terms of clinical diagnosis and neuropsychological tests. *Aβ* β-amyloid, *ADAS-Cog13* 13-item version of the Alzheimer’s Disease Assessment Scale-Cognitive Subscale, *ADNI* Alzheimer’s Disease Neuroimaging Initiative, *ADNI MEM* ADNI episodic composite memory score, *CU* cognitively unimpaired, *Diff*. difference, *MCI* mild cognitive impairment, *MMSE* Mini Mental State Examination, *mon* months, *PACC* Preclinical Alzheimer Cognitive Composite score, *PET* positron emission tomography, *T* time].

ADNI3 has been conducted in full conformity with Good Clinical Practice guidelines and Regulations for the Protection of Human Subjects of Research. The ADNI3 protocol complies with the International Conference on Harmonization, Health Insurance Portability and Accountability Act, and State and Federal regulations, and was approved by local Institutional Review Boards (IRB). Individuals provided written consent to participate with IRB-approved informed consent forms. A complete listing of ADNI investigators and participating sites can be found at the end of the article.

### PET imaging biomarkers

Aβ PET (18F-florbetapir or 18F-florbetaben) scans were rated as either Aβ(+) (summary standardized uptake value ratio (SUVR) ≥ 1.11 [29, 30] or 1.08 [31], respectively) or negative (Aβ(-)), based on the uptake of tracer in a cortical summary region of interest (ROI) with previously validated tracer-specific cut-offs, relatively to the uptake in the whole cerebellum (reference region). The Aβ load measured in centiloids (CL) was also assessed [32, 33].

**Fig. 2:**
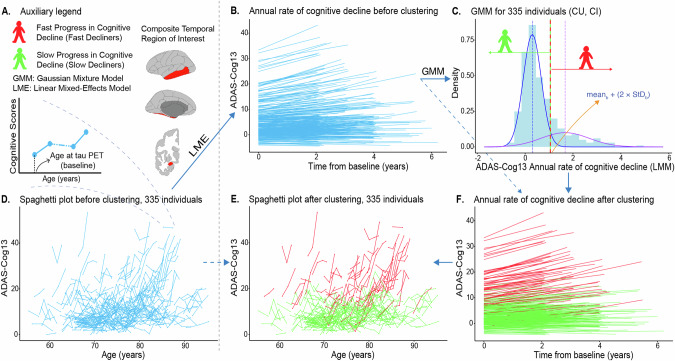
The clustering pipeline for the definition of SDs and FDs in the case of the ADAS-Cog13 score. **A** Auxiliary legend for the rest of the analysis. The composite temporal region of interest illustrates the regions that make up the temporal meta-ROI according to the ADNI3 protocol (bilateral amygdala, entorhinal, fusiform, inferior, and middle temporal cortices). **B** The LME outcome for the definition of the annual rate of cognitive decline. The slope of each line represents the annual rate of cognitive decline for each individual. Longitudinal data (≥2 years after the baseline examination) for the ADAS-Cog13 were available for 338 out of the 396 individuals, but three individuals were excluded as outliers/influential points. Therefore, our analysis pipeline was completed for 335 individuals. **C** The GMM outcome for clustering the individuals as SDs or FDs. The distribution of the annual rates of cognitive decline can be explained by two normal distributions (blue: mean_b_ = 0.298, StD_b_ = 0.378; purple: mean_p_ = 1.669, StD_p_ = 1.005). Individuals with an annual rate of cognitive decline ≥ mean_b_ + (2 × StD_b_) ≈1.05 were clustered as FDs. **D**, **E** Spaghetti plots representing all the ADAS-Cog13 scores for each individual before and after clustering respectively. **F** Similar to B, but after clustering. ADAS-Cog13 13-item version of the Alzheimer’s Disease Assessment Scale-Cognitive Subscale, ADNI Alzheimer’s Disease Neuroimaging Initiative, CI cognitively impaired, CU cognitively unimpaired, FD fast decliner, SD slow decliner, GMM Gaussian mixture model, LME linear mixed-effects model, ROI region of interest, StD standard deviation.

For tau PET, the uptake of 18F-flortaucipir (aka Tauvid, AV-1451, T807) was quantified using SUVR relative to the uptake in the inferior cerebellar grey matter for all cortical ROIs. 18F-flortaucipir is a reliable biomarker for advanced tau pathology (Braak stages V-VI) [34, 35]. To summarise the tracer uptake, we used a standard composite temporal ROI (temporal meta-ROI) (Fig. 2A) [36, 37]. Tau PET positivity (T(+)) was tested in a pilot manner with a previously suggested threshold (1.34 SUVR in the temporal meta-ROI) [38], derived from the largest independent cohort to date. A gray zone of 5% was used to compensate for the absence of postmortem validation for the definition of T(+). To assess the applicability of the published threshold in our cohort, we followed the previously reported methodology [38, 39]. A very similar cut-off value was calculated (Supplement; Methods, Supplementary Fig. 1). The ADNI pipeline was used for preprocessing the Aβ and tau PET imaging data [17]. The results after applying the correction for partial volume effects (PVC) are presented in the Supplement as detailed below.

FDG PET scans were extracted from ADNI and were visually interpreted in cases of accelerated cognitive decline, by two independent nuclear medicine specialists blinded to the individual Aβ and tau biomarker status. The assessment of the FDG PET scans was assisted by the Syngo.via (Siemens Healthcare), an FDA-approved software platform [40]. The scans were dichotomously categorized as either suggestive of AD or not suggestive of AD (i.e., showing no specific neurodegeneration pattern or a pattern suggestive of another neurodegenerative disorder).

### Cognitive measures

The Mini Mental State Examination (MMSE) was only used to characterize the global cognition of individuals at baseline. The 13-item version of the Alzheimer’s Disease Assessment Scale-Cognitive Subscale (ADAS-Cog13) and the Preclinical Alzheimer Cognitive Composite (PACC) score were used as measures of global cognition over the multiple time points of the follow-up interval [41, 42]. Cognitive impairment in episodic memory was measured using the ADNI episodic composite memory score (ADNI MEM) [43].

### Comorbidities

Information regarding the medical history at study enrollment (i.e., cerebrovascular disease risk factors, psychiatric or neurological comorbidities) [44, 45], and the use of medications at every visit, has been recorded at the ADNI depository and was manually assessed by two independent medically trained investigators (KI, KC) (Supplementary Table 1). The vascular risk factors (VRF) burden (Table 2) was defined as previously detailed [46].

**Table 2:** Clinical characteristics of Aβ(+) fast decliners by tau PET status.

Aβ/Tau PET status	Aβ(+) Fast decliners		*p*-values^b^
	Aβ(+)T(-) *(N* = *19)*^a^	Aβ(+)T(+) *(N* = *34)*^a^	
**Baseline**			
**Age, y**	77.5 (6.87)	75.57 (7.23)	0.4
**Sex**			0.8
Male	10 (53%)	16 (47%)	
Female	9 (47%)	18 (53%)	
**Education, y**	15.79 (3.05)	16.24 (2.44)	0.6
**Cognitive status**			0.7
CU	4 (21%)	5 (15%)	
CI	15 (79%)	29 (85%)	
**ADAS-Cog13**	17.17 (8.69)	22.75 (10.09)	**0.044** *
**APOE4 (**≥**1 allele)**	10/19 (53%)	20/31 (65%)	0.6
Not available	0	3	
**Centiloids at Aβ PET**	63.67 (26.75)	83.03 (31.42)	**0.038** *
**Tau PET SUVR (temporal meta-ROI)**	1.23 (0.07)	1.58 (0.23)	**<0.01** **
**FDG PET hypometabolism pattern**			**0.039** *
Suggestive of AD	2/14 (14%)	12/24 (50%)	
Not suggestive of AD	12/14 (86%)	12/24 (50%)	
Not available	5	10	
**Follow-up**			
**Interval, mon**	34.56 (9.48)	34.56 (10.92)	0.8
**ADAS-Cog13 annual decline rate**	2.06 (0.93)	2.19 (0.8)	0.4
**Medical history and medication use**^c^			
**Cerebrovascular disease risk factors**			
Antiplatelets/Anticoagulants	13 (68%)	22 (65%)	>0.9
Hyperlipidemia/Antihyperlipidemic agents	15 (79%)	18 (53%)	0.08
Hypertension/Antihypertensive agents	13 (68%)	21 (62%)	0.8
History of cardiovascular disease	5 (26%)	7 (21%)	0.7
Smoking	5 (26%)	5 (15%)	0.5
Nitrates	2 (11%)	5 (15%)	>0.9
Diabetes mellitus/Antidiabetic agents	2 (11%)	2 (5.9%)	0.6
Atrial fibrillation/Flutter	0 (0%)	4 (12%)	0.3
Stroke/TIA	0 (0%)	3 (8.8%)	0.5
VRF burden (yes/no)^d^	13 (68%)	17 (50%)	0.3
**Psychiatric comorbidities**			
Psychiatric comorbidity	10 (53%)	9 (26%)	0.077
History of depression (≥1 episodes)	9 (47%)	6 (18%)	**0.029** *
Antidepressant agents	11 (58%)	12 (35%)	0.2
Depression ongoing	9 (47%)	5 (15%)	**0.021** *
Depression ongoing (>1 episodes)	8 (42%)	5 (15%)	**0.044** *
GDS (short form)	3.05 (2.74)	2.29 (2.69)	0.2
Other antipsychiatric agents	4 (21%)	4 (12%)	0.4
Anxiety disorder	3 (16%)	5 (15%)	>0.9
**Other**			
History of major CNS disorder	1 (5.3%)	2 (5.9%)	>0.9
Medication for AD (i.e., acetylcholinesterase inhibitors, Memantine)	12 (63%)	17 (50%)	0.4

^a^Mean (Standard deviation); *n/N* (%).

^b^Kruskal-Wallis rank sum test; Fisher’s exact test.

^c^Comorbidities were assessed for all individuals at the beginning of ADNI3; medications were assessed at enrollment and during the ADNI3 phase. The term psychiatric comorbidity includes individuals who had at least one psychiatric disorder; this includes all categories of depression and anxiety disorder and one individual [CU, Aβ(+)T(+)FD] who was diagnosed with seasonal affective disorder.

^d^The presence of VRF burden was defined as the coexistence of two or more of the following conditions: (i) cardiovascular disease, (ii) hypertension (positive medical history or use of antihypertensive medication), (iii) diabetes mellitus (positive medical history or use of antidiabetic medication), (iv) hyperlipidemia (positive medical history or use of antihyperlipidemic medication), (v) stroke or TIA, (vi) smoking (ever or never), (vii) atrial fibrillation, and (viii) left ventricular hypertrophy. *Aβ* β-amyloid, *ADNI* Alzheimer’s Disease Neuroimaging Initiative, *CI* cognitively impaired, CU cognitively unimpaired, *CNS* central nervous system, *FD* fast decliner, *GDS* geriatric depression scale, *T* tau, *TIA* transient ischemic attack, *VRF* vascular risk factors].

### Statistical analysis

The heterogeneity of cognitive impairment trajectories was assessed over the follow-up interval in terms of both clinical conversion (i.e., change of diagnosis) and annual rate of cognitive decline (fast (FDs) vs. slow decliners (SDs)) (Fig. 1). The level of statistical significance was set at α = 0.05. Supplementary Table 2 summarizes the rationale of our analysis.

#### Progress in diagnosis

All CU and MCI individuals were divided into two groups according to their baseline and last follow-up diagnoses. The individuals who retained their initial diagnosis (i.e., CU to CU, MCI to MCI) were classified as Non-Converters, while those progressing in clinical severity were classified as Converters (i.e., CU to MCI, CU/MCI to dementia). Individuals with a dementia diagnosis at baseline were excluded from this analysis. The two groups were compared in terms of baseline tau PET uptake descriptively. The prognostic accuracy of both baseline Aβ and tau PET uptake in predicting clinical conversion was evaluated using receiver operating characteristic (ROC) analysis. Baseline Aβ and tau PET were also independently assessed in terms of sensitivity (Se), specificity (Sp), positive predictive value (PPV), and negative predictive value (NPV).

#### Progress in cognitive decline

Longitudinal cognitive performance was modelled to assess individuals’ annual rate of cognitive decline using separate linear mixed-effects (LME) models for each neuropsychological test. Akaike information criterion (AIC) was used for model selection. The time from baseline was set as a fixed and random effect to allow for the heterogeneity in cognitive trajectories across individuals. A random intercept was included to capture differences in baseline cognitive performance across individuals. Age at baseline and years of education were added as covariates to the models. The contribution of sex was not significant and did not contribute substantially to the model. Using influence plots and measures of Cook’s distance, we identified and excluded outliers and influential points as documented in the respective figure legends [47, 48].

Gaussian mixture models (GMM) were used for identifying, in a probabilistic manner, the number of underlying normal distributions that best explained the distribution of annual rates of cognitive decline for each neuropsychological test; these were extracted from the LME models of the previous step. In all neuropsychological tests, the annual rate of cognitive decline was best modelled by two normal distributions. The mean and the standard deviation (StD) of the distribution with the lower annual rates of cognitive decline were used to define a threshold (∣mean + (2 × StD)∣). Individuals with an annual rate of cognitive decline greater than or equal to the threshold value were clustered as FDs, while the others were clustered as SDs (Fig. 2). The tau PET uptake was descriptively compared across these two groups. The prognostic accuracy of both baseline Aβ and tau PET uptake in predicting fast cognitive decline (FDs) was evaluated using ROC analysis. Se, Sp, PPV, and NPV of both baseline Aβ and tau PET were also independently assessed.

#### Baseline tau PET uptake and rate of cognitive decline

We assessed the association between baseline tau PET uptake and the annual rate of cognitive decline with linear modelling. AIC was used for model selection. The temporal meta-ROI SUVR, the Aβ PET status (Aβ(-/+)), the baseline cognitive status (CU/CI), and the interaction between temporal meta-ROI SUVR and Aβ PET status were set as predictors of the annual rate of cognitive decline. The estimated marginal means of the linear model were used to perform post hoc comparisons on the interaction term. The existence of outliers and influential points was tested for, as detailed above. The risk ratio (RR) for being Aβ(+)FD in relation to the tau PET status (T(+) vs. T(-)) was evaluated.

#### Comorbidities and FDG PET hypometabolism patterns

The prevalence of non-AD comorbidities and FDG PET hypometabolism patterns in cases of accelerated cognitive decline was evaluated and compared with Fisher’s test (Aβ(+)T(–)FDs vs. Aβ(+)T(+)FDs), both including and excluding (Supplementary) the individuals located in the gray zones defined for T(–/+) and SD/FD.

#### Software

R version 4.1.1 was used for statistical analysis (Supplementary Table 1).

## Results

We identified 396 individuals from the ADNI dataset with baseline Aβ and tau PET scans who had completed a ≥ 2-year follow-up (Table 1).

### Progress in diagnosis

When analyzed separately, the accuracy (area under the curve; AUC) of baseline Aβ and tau PET uptake for predicting clinical conversion was AUC = 0.74 and AUC = 0.72, respectively (Supplementary Fig. 2A). Aβ PET showed higher Se = 0.72 and NPV = 0.94, compared to tau PET but the latter showed higher Sp = 0.92 and PPV = 0.44 (Supplementary Table 3).

**Fig. 3:**
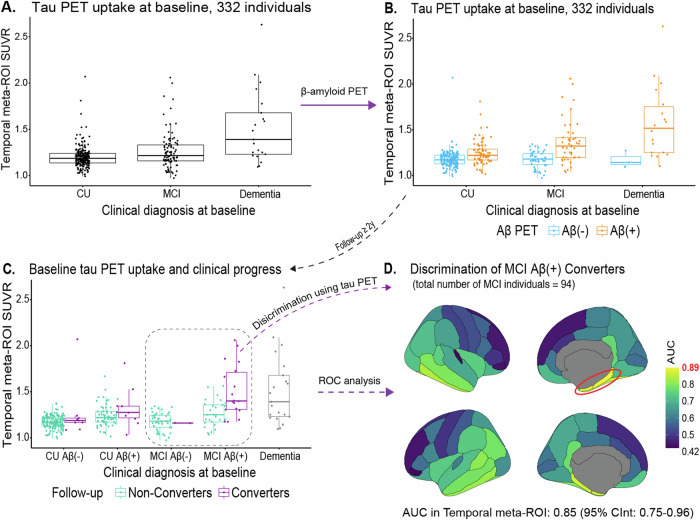
Baseline tau PET uptake and clinical conversion. **A**, **B** Baseline tau PET uptake with respect to clinical and biomarker diagnoses, respectively. **C** Baseline tau PET uptake in relation to the follow-up status per diagnostic group. **D** The results of a ROC analysis illustrated in a brain atlas for the discrimination of MCI Aβ(+) Converters among all MCI individuals. Aβ β-amyloid, AUC area under the curve, CInt confidence interval, CU cognitively unimpaired, MCI mild cognitive impairment, PET positron emission tomography, ROC receiver operating characteristic, ROI region of interest; SUVR standardized uptake value ratio.

MCI Aβ(+) Converters (*n* = 18, 39%, Table 1) were characterized by higher baseline tau PET uptake than the other groups (Fig. 3). The accuracy of tau PET uptake in predicting MCI Αβ(+) Converters was AUC = 0.85 in the temporal meta-ROI. The entorhinal and the parahippocampal cortices showed the highest AUC value (AUC = 0.89) (Fig. 3D, Supplementary Fig. 3A).

The accuracy of baseline tau PET uptake in predicting clinical conversion in the CU group was not evaluated separately, given that the number of CU individuals who converted to MCI or dementia (*n* = 9, 6% in Aβ(-) and *n* = 10, 14% in Aβ(+) individuals, Table 1) was not large enough to draw reliable conclusions.

### Progress in cognitive decline

An annual rate of cognitive decline of 1.05 units in the ADAS-Cog13 score was set as the threshold for defining FDs (Fig. 2C). When analyzed separately, the accuracy of baseline Aβ and tau PET uptake for predicting FDs was AUC = 0.74 and AUC = 0.78, respectively (Supplementary Fig. 2B). The Se = 0.76 and NPV = 0.91 of Aβ PET were higher compared to tau PET, even though the NPV = 0.87 of tau PET was similarly high. The latter also showed higher Sp = 0.94 and PPV = 0.69 (Supplementary Table 3).

**Fig. 4:**
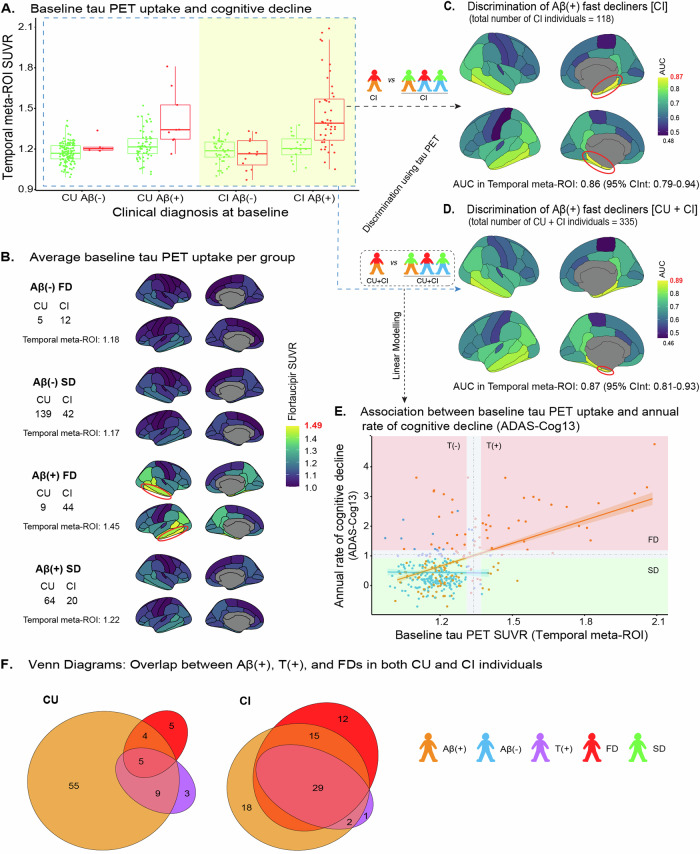
Baseline tau PET uptake and progress in cognitive decline. **A** Baseline tau PET uptake with respect to SD/FD profiles in both CU and CI individuals. **B** Average baseline tau PET uptake per group according to Aβ(-/+) and SD/FD profiles. **C**, **D** The results of a ROC analysis illustrated in brain atlases for the discrimination of Aβ(+)FDs among CI and CU + CI individuals, respectively. **E** Association (linear modelling) between the baseline tau PET uptake and the annual rate of cognitive decline including information about both Aβ(-/+) and SD/FD. FD was defined as ADAS-Cog13 annual rate of cognitive decline ≥ 1.05; the red/green shaded areas depict FD/SD; the gray zone for the ADAS-Cog13 annual rate of cognitive decline was obtained from mean_b_ + [(1.65 – 2.35) × StD_b_] ≈ (0.92–1.19) (Fig. 2C). T(+) was defined as tau PET SUVR ≥ 1.34 in the temporal meta-ROI; the gray zone for tau PET was obtained from 1.34 ± (2.5% × 1.34) ≈ (1.31–1.37). No outliers/influential points were found. **F** Venn diagrams to illustrate the overlap of Aβ(+), T(+), and FDs in both CU and CI individuals. The size of each group is depicted proportional to the number of individuals it comprises. Aβ β-amyloid, ADAS-Cog13 13-item version of the Alzheimer’s Disease Assessment Scale-Cognitive Subscale, AUC area under the curve, CInt confidence interval, CI cognitively impaired, CU cognitively unimpaired, FD fast decliner, SD slow decliner, PET positron emission tomography, ROC receiver operating characteristic, ROI region of interest, SUVR standardized uptake value ratio, T tau.

Baseline tau PET uptake was higher in Aβ(+)FDs, either CU or CI, than in Aβ(-)FD/SDs and Aβ(+)SDs in the temporal meta-ROI (Fig. 4A–B). The accuracy of tau PET uptake in predicting Aβ(+)FDs was AUC = 0.86 in the CI group separately, and AUC = 0.87 considering all individuals (Fig. 4C, D, Supplementary Fig. 3B–C) (AUC = 0.90 in the PVC data, Supplementary Fig. 4). The corresponding AUC values, for both these scenarios, for the cortical ROIs composing the temporal meta-ROI varied between 0.81 and 0.89, when analyzed separately, whereas cortical ROIs beyond them (e.g. inferior parietal cortex), showed lower AUC (Fig. 4C, D, Supplementary Fig. 5). Baseline tau PET uptake was differentially linearly related to the annual rate of cognitive decline in Aβ(+) and Aβ(-) individuals (interaction t = 4.275, *p* < 0.001); the association was statistically significant in Aβ(+) individuals (95% confidence interval 2.07–3.09), but not in Aβ(-) individuals (95% confidence interval -1.19–1.04) (Fig. 4E). The results for the PACC score and ADNI MEM are reported in Supplementary Fig.s 4, 6.

### Overlap of Aβ(+), T(+) and fast cognitive decline

T(+) individuals had 10 times the risk of being Aβ(+)FDs compared to T(-) individuals (RR = 10.4, *p* < 0.001, 95% confidence interval 6.5–16.8). Based on the group intersections, T(+) individuals constituted largely a subgroup of Aβ(+)FDs (Aβ(+)T(+)FDs). The overlap between Aβ(+), T(+) and FDs was more prominent in CI individuals. Most FDs (*n* = 70) were Aβ(+)T(+) (*n* = 34, 49%) or, less commonly, Aβ(+)T(-) (*n* = 19, 27%). No Aβ(-)T(+)FDs were identified. Only three CU individuals and one CI individual showed isolated tau positivity and were SDs (Aβ(-)T(+)SDs) (Fig. 4E, F).

### Aβ(+)T(-)FDs vs. Aβ(+)T(+)FDs

#### Clinical characteristics and comorbidities

No statistically significant differences were observed between Aβ(+)T(-)FDs and Aβ(+)T(+)FDs with respect to demographics and the prevalence of APOE4 allele (Table 2, Supplementary Table 4, Supplementary Fig. 7). Baseline ADAS-Cog13 was higher in Aβ(+)T(+)FDs (*M* = 22.75, StD = 10.09) than in Aβ(+)T(-)FDs (*M* = 17.17, StD = 8.69) (*p*-value = 0.044), but not the annual rate of decline in the same test (*p*-value = 0.4). A higher baseline Aβ PET load was also detected in Aβ(+)T(+)FDs (*M* = 83.03CL, StD = 31.42) compared to Aβ(+)T(-)FDs (*M* = 63.67CL, StD = 26.75) (*p*-value = 0.038). The prevalence of cerebrovascular disease risk factors and psychiatric comorbidities was higher in Aβ(+)T(-)FDs than in Aβ(+)T(+)FDs, although only the prevalence of depression diagnosis reached statistical significance (47% vs. 15%, *p*-value = 0.021) (Table 2, Supplementary Fig. 8). The results for separate analysis of CI individuals are shown in Supplementary Fig. 9.

#### FDG PET hypometabolism pattern

**Fig. 5:**
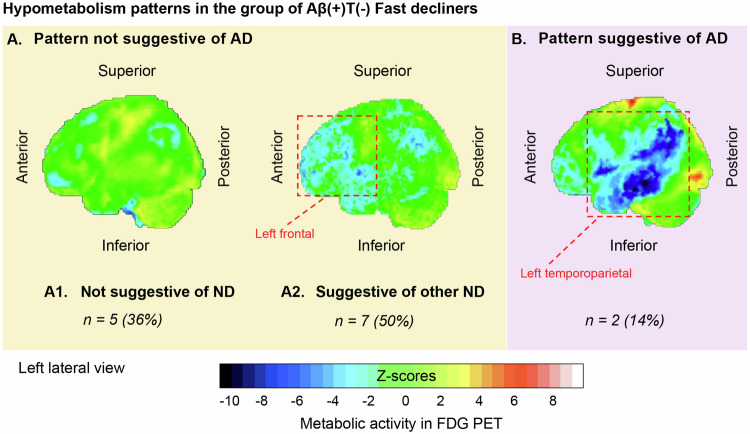
Hypometabolism FDG PET patterns in the group of Aβ(+)T(-) fast decliners (14 out of 19 individuals had available FDG PET scans). A representative FDG PET scan from each category of hypometabolism pattern that we identified in this group is presented. The dashed red line encloses regions with hypometabolism. **A** Hypometabolism pattern not suggestive of AD (A1 + A2, *n* = 12 (86%)). **B** Hypometabolism pattern suggestive of AD (*n* = 2 (14%)). The analysis of the FDG PET images was made in an FDA-approved software platform i.e., Syngo.via (Siemens Healthcare) (Wilson, Selwyn, and Elojeimy 2022); 3D analysis in stereotactic surface projection. The Z-scores scale is based on the Syngo.via database that consists of healthy volunteers aged 46 to 79 years old and the whole brain has been used for intensity normalization [(statistic = (Value_patient_ – Mean_population_)/Std.Dev_population_]. The resulting images were visually assessed by two independent nuclear medicine specialists. AD Alzheimer’s disease, Aβ β-amyloid, Aβ(+) Aβ positivity, FDA food and drug administration, FDG 18F-fluorodeoxyglucose, ND neurodegenerative disorder, PET positron emission tomography, T(-) tau negativity.

Out of 53 Aβ(+)FDs, 38 had available baseline FDG PET scans for visual assessment. The hypometabolism pattern was different between Aβ(+)T(-)FDs and Aβ(+)T(+)FDs (*p*-value = 0.039). Most Aβ(+)T(-)FDs showed a hypometabolism pattern not suggestive of AD (*n* = 12, 86% vs. *n* = 2, 14%), whereas 50% of Aβ(+)T(+)FDs (*n* = 12) showed a hypometabolism pattern suggestive of AD (Table 2). Out of 12 (86%) Aβ(+)T(-)FDs who showed a hypometabolism pattern not suggestive of AD, 5 individuals (36% of Aβ(+)T(-)FDs) showed no specific neurodegeneration pattern in FDG PET, and 7 individuals (50% of Aβ(+)T(-)FDs) showed a pattern suggestive of another neurodegenerative disorder (Fig. 5).

### Aβ(-)T(-)FDs

Aβ(-)T(-)FDs showed high prevalence of non-AD comorbidities (i.e., depression diagnosis; *n* = 5, 29%). Most Aβ(-)T(-)FDs (*n* = 9, 69%) also exhibited a hypometabolism pattern not suggestive of AD (Supplementary Table 5).

### Follow-up tau PET scans in Aβ(+)T(-)FDs and Aβ(-)T(-)FDs

Nine out of 19 Aβ(+)T(-)FDs and six out of 17 Aβ(-)T(-)FDs had available follow-up tau PET scans. All individuals remained T(-) (Supplementary Table 6).

## Discussion

Baseline tau PET uptake determined with high accuracy (AUC = 0.85–0.87) the subset of Aβ(+) individuals who has experienced prospective disease progression in terms of conversion of baseline diagnosis or accelerated cognitive decline. Our findings confirm the results of previous studies [19, 20] and provide further support for the ideas that: 1.) the buildup of Aβ pathology is much more prevalent among even CU elderly than that of extensive tau pathology detected with tau PET (Braak stages V-VI), which explains the suboptimal accuracy of Aβ biomarkers to predict deterioration [7–9, 34, 49, 50], 2.) T(+) individuals constitute largely a subset of those being Aβ(+) in AD cohorts [20], 3.) the presence of tau exceeding Braak IV – above the PET detection limit – greatly increases the risk for AD-relevant cognitive decline [15, 17, 18, 51]. We further present novel findings indicating that fast cognitive decline in individuals with isolated Aβ positivity is associated with a hypometabolism pattern not suggestive of AD and a high prevalence of depression diagnosis. This implies that in cases of isolated Aβ positivity, cognitive decline may be associated with changes within the AD spectrum in a multi-morbidity context (mixed AD) [52–54].

Interestingly, our study highlighted the presence of cases of Aβ(+) individuals with fast cognitive decline but a negative tau PET scan. The assessment of cognitive decline when accompanied by Aβ and tau discordance, or alternatively, of the individuals that may be overlooked if prioritizing tau over Aβ biomarkers, is a pivotal step before the clinical establishment of tau biomarkers. The interpretation of isolated Aβ positivity is quite challenging and two scenarios might explain the discordance (Aβ(+)T(-)) in the context of prospective deterioration. These individuals would either still have sufficient tau pathology to qualify for AD but we cannot detect it because of the limited sensitivity of tau PET to detect early tau pathology (i.e., false negative cases that could represent AD), or other pathologies could have been present contributing to cognitive decline by acting synergistically or additively to Aβ pathology [11, 46, 55, 56]. The first scenario cannot be excluded given the limited available postmortem data for individuals with antemortem tau PET data. The first scenario aligns with the lower baseline ADAS-Cog13 we observed in individuals exhibiting fast cognitive decline and isolated Aβ positivity (Aβ(+)T(-)FDs) compared to individuals presenting with combined evidence of Aβ and tau pathologies (Aβ(+)T(+)FDs) (Table 2). This concept implies that the former may be on the AD pathway but at an earlier stage [57]. On the other hand, the second scenario would be consistent with 1.) the high prevalence of mixed pathologies observed in autopsy studies [58, 59], where the total burden of pathology could be the determinant for cognitive decline [53, 54], and 2.) the finding that Aβ pathology has a high prevalence even in cases where a non-AD diagnosis is most likely [11, 12]. Indeed, Aβ(+)T(-)FDs in our study showed a significantly higher prevalence of depression, significantly lower average levels of Aβ pathology, and a higher prevalence of a hypometabolism pattern not suggestive of AD compared to Aβ(+)T(+)FDs (Table 2). The latter aligns with the scenario of multimorbidity in the Aβ(+)T(-)FDs and highlights the heterogeneity among Aβ(+) individuals [57, 60, 61], which reinforces the argument that cognitive decline associated with isolated Aβ positivity should be cautiously interpreted.

Taken together, our data support the hypothesis that tau positivity allows discrimination of individuals with AD-relevant cognitive decline and suggest that the consideration of incorporating tau PET in tertiary memory clinics could be advantageous since it offers both diagnostic information (45/49 (92%) of T(+) individuals were Αβ(+)) and prognostic insight (since T(+) individuals followed a fast trajectory of cognitive decline). Nevertheless, our findings cannot provide definite evidence for or against the hypothesis that when biomarker status is discordant, the underlying condition driving decline is not AD-related. The complex coincident nature of AD and non-AD comorbidities [45, 46, 62, 63] underlines the need for further research in individuals with discordant biomarker status before one suggests the prioritization of tau over Aβ biomarkers, as a standalone biomarker strategy. It is important to consider that the clinical applicability of a biomarker depends on its characteristics and different properties are required based on its desired clinical utility [64, 65].

The novelty of our study arises from the effort to understand the biomarkers discordance in the setting of multimorbidity and from the methodological approach we followed for determining clusters of individuals based on their prospective cognitive performance. The role of mixed pathologies in cognitive decline is common knowledge [52–54, 60, 61]. However, this is one of the few studies that took into consideration both the prevalence of non-AD comorbidities and the FDG PET hypometabolism pattern in the context of interpreting isolated Aβ positivity and establishing the clinical applicability of tau biomarkers. Our study provides proof-of-concept evidence that in the case of Aβ and tau discordance (Aβ(+)T(-)), cognitive decline could be associated with changes within the AD spectrum in a multi-morbidity context, i.e., mixed AD, while in the case of Aβ and tau positivity concordance (Aβ(+)T(+)), AD pathology is the main contributor to cognitive decline. This could facilitate the development of individualized medicine and offer novel insights into: 1) the role of comorbidities when interpreting discordant AD biomarkers and the outcomes of clinical trials; and 2) the potential of a standalone tau biomarker strategy in clinical routine. We are not aware of other previous studies testing this hypothesis.

In contrast to prior research efforts which focused solely on clinical conversion to define disease progression, i.e., change of diagnosis over time, or employed cognition as a continuous measure, we clustered the individuals based on their longitudinal cognitive performance. Although machine learning techniques and multiple cognitive scores have also been previously used to define clusters of decliners, these approaches possess inherent weaknesses and lack clinical interpretation [27, 66, 67]. To overcome these limitations, we used longitudinal modelling for assessing the annual rate of cognitive decline and a data-driven approach for clustering individuals in an unbiased manner as FDs or SDs [68]. This methodology has substantial advantages over the aforementioned approaches and provides more powerful data for detecting clinically relevant cognitive decline. A diagnosis of clinical conversion can depend on both the operator (i.e., different physicians) and the information provided by the individual’s study partner [69–71]. Additionally, the investigation of clinical conversion cannot assess progression in patients with dementia at baseline since they cannot progress more in terms of diagnosis. Our modelling approach for longitudinal cognitive trajectories also allows adjustment for relevant covariates and is more powerful in shorter follow-up intervals. Furthermore, the clustering of individuals into SDs and FDs rather than using cognition as a continuous variable [17] allowed us to assess the prognostic accuracy of the biomarker and, therefore, its clinical applicability to the individual level. While it is difficult to determine whether our clustering approach is optimal for defining individuals with clinically relevant cognitive decline – this construct could be controversial – similar data-driven approaches have been used previously in clinical trials [72] and they are powerful in detecting a minimal clinically important difference in longitudinal data [68]. We are confident that our findings hold since a.) the follow-up interval we employed aligns with that used in other recent pertinent studies [19, 20] and clinical trials [73] and has previously been suggested as optimal for evaluating the prognostic accuracy of AD biomarkers [74], and b.) the threshold we evaluated for defining fast cognitive decline is consistent with the existing literature [75].

The study also yielded unanticipated results. Firstly, the areas where tau PET uptake was most accurate in predicting disease progression were restricted to within the medial temporal lobe (i.e., entorhinal cortex) (Fig. 4C, D, Supplementary Fig. 5). Evidence from autopsy studies has suggested that isolated tau accumulation in the medial temporal lobe is a common finding in CU elderly [50] and is not associated with a neuropathological AD diagnosis even in the presence of Aβ pathology [76]. Historically, only the spread of tau pathology into more widespread brain areas has been associated with cognitive decline [51]. This contradiction between our in vivo and earlier pathological evidence could be explained by the facts that 1) 18F-flortaucipir detects only advanced tau pathology (i.e., Braak stages V-VI), while at autopsy the whole spectrum, burden, topography, and packing density of tau pathology are revealed and assessed; and 2) the burden of tau pathology in the entorhinal cortex, and therefore the tau PET tracer uptake, increases from Braak stage I to VI [34]. Secondly, regarding the clustering into SDs and FDs, while some argue that the ADAS-Cog lacks sensitivity in detecting early cognitive decline [77], we found that it yielded similar results to those based on the PACC score, and the ADNI MEM [41, 42] with respect to the evaluated prognostic accuracy of tau PET (Supplementary Fig.s 4, 6). Thirdly, cases of Aβ and tau positivity accompanied by slow cognitive decline (Aβ(+)T(+)SDs) were also observed (*n* = 11, 3.28%), representing individuals probably earlier in the disease course or those with high cognitive resilience (Fig. 4F, Supplementary Table 7).

The extrapolation of our findings is subject to certain limitations with respect to a.) the characteristics of ADNI participants, b.) the follow-up interval, c.) our methodological approach regarding the longitudinal modeling of cognitive decline and the clustering into groups of decliners, and d.) the interpretation of PET imaging data. ADNI has been designed as an AD cohort and individuals with clear evidence of other major comorbidities have been excluded, limiting the interpretation of our findings to this cohort. Although we consider the sample size adequate for the analysis when grouping CU and CI individuals together, it does not guarantee a high level of confidence in analyses within specific subgroups. More specifically, the power could be limited in CU individuals since the annual conversion rate in this group was low, consistent with previously reported rates (5–10%) [78–80], and the percentage of FDs was only 6% (14/217). This precludes any certain conclusions about the utility of the biomarker in CU highlighting the power issues that emerge when studying this population. Similarly, the small number of Aβ(+)T(-)FDs limits the confidence of our findings concerning the role of non-AD comorbidities and the prevalence of FDG PET hypometabolism patterns when cognitive decline is associated with isolated Aβ positivity. The follow-up interval was limited to an average of 33.7 months (in the case of the ADAS-Cog13, Table 1). Although clinically relevant cognitive decline is generally detected within this interval, the wide range of individual-level follow-up intervals (24–74.7 months) may also have biased the modeling of cognitive decline, especially in individuals with shorter intervals. Earlier studies have also shown that cognitive decline follows a non-linear trajectory [28, 81], and especially individuals at earlier stages of cognitive decline might need longer follow-up intervals to reach the turning point into accelerated cognitive decline [82–84]. However, given the limited follow-up interval and time-points, we restricted our analysis to linear models, which could have led to an underestimation of the annual rate of decline, especially in CI individuals. We should also keep in mind that while beneficial with respect to clinical applicability, clustering individuals solely based on cognitive performance may introduce some degree of generalization and overlook important idiosyncratic characteristics. The limitations pertaining to the PET data concern a.) the limited sensitivity of 18F-flortaucipir for detecting early tau pathology (i.e., Braak stages I-IV) [34, 35], and b.) the dichotomous interpretation of Aβ and FDG PET scans, which might oversimplify the information that can be obtained from these biomarkers. Briefly, although quantitative Aβ and FDG PET show associations with cognitive decline [85–89], we selected a dichotomous approach to align more with the interpretation of biomarkers in a clinical setting [64, 90–92].

Further research in other cohorts, with longer follow-up intervals, and employing different tau PET tracers [93, 94] is needed to validate our results and explore differences among cognitive tests, especially in CU individuals. Future real-world clinical data, with available postmortem evaluation, could also help us investigate the pathological correlates of the underexplored and relatively rare isolated tau positivity (Aβ(-)T(+), 1.19% of our ADNI sample) and better understand the role of comorbidities in individuals with clinically relevant cognitive decline and discordant Aβ and tau status. Finally, the visual read of tau PET scans is also needed to assess both the prevalence and the significance of atypical patterns of tau propagation that might go undetected when using thresholds and composite ROIs.

## Conclusions

Tau PET imaging showed high accuracy to predict the subset of Aβ(+) individuals that will show AD-relevant cognitive decline. We provided proof-of-concept evidence that, when accelerated cognitive decline is associated with isolated Aβ positivity, the Aβ load falls within the low-positive range, the hypometabolism pattern is often not suggestive of AD, and the prevalence of depression diagnosis is high. These factors collectively suggest that cognitive decline accompanied by isolated Aβ positivity may be associated with changes within the AD spectrum in a multi-morbidity context, i.e., mixed AD. This should be investigated further before prioritizing tau over Aβ biomarkers. Overall, tau PET can predict a population of high clinical interest and should be considered as a combined diagnostic and prognostic tool with both clinical and research applications for the management of cognitively impaired individuals.

## Supplementary information

Supplement

## Data Availability

The datasets generated during and/or analysed during the current study are available in the ADNI repository, https://ida.loni.usc.edu/login.jsp?project=ADNI.

## Consortia

Data used in preparation of this article were obtained from the Alzheimer’s Disease Neuroimaging Initiative (ADNI) database (adni.loni.usc.edu). As such, the investigators within the ADNI contributed to the design and implementation of ADNI and/or provided data but did not participate in analysis or writing of this report. A complete listing of ADNI investigators can be found at: https://adni.loni.usc.edu/wp-content/uploads/how_to_apply/ADNI_Acknowledgement_List.pdf.
