# Supplementary material for: Tau PET positivity predicts clinically relevant cognitive decline driven by Alzheimer’s disease compared to comorbid cases; proof of concept in the ADNI study
Source: Mol Psychiatry. 2024 Aug 23;30(2):587–99. doi: 10.1038/s41380-024-02672-9 (PMC11746147; doi:10.1038/s41380-024-02672-9)
Supplement: Supplementary file 1 — Supplement [file 41380_2024_2672_MOESM1_ESM.docx]

**Supplementary Material**

**Methods**

About the tau PET threshold applicability

To test the applicability of a previously published tau PET threshold for the temporal meta-ROI (1.34) in our sample, we followed the same methodology as reported by Ossenkoppele R, et al. 2018. We evaluated a tau PET threshold based on an independent sample of CU individuals from the ADNI cohort that did not meet the inclusion criteria of the present study.

We first identified all the available tau PET scans from the ADNI dataset (n = 1304). In order to avoid the selection-bias in case that one individual had undergone more than one tau PET scans, we selected the baseline (first) tau PET scan of each individual (n = 837). Next, we evaluated the threshold for tau PET positivity [T(+)] based on the distribution of tau PET SUVR values in the temporal meta-ROI of 225 CU individuals, not included in the sample of 335 individuals that completed our analysis pipeline, as detailed in the main text, for the ADAS-Cog13 score (ADAS-Cog13 sample), using the mean + (2 × StD) approach (Supplementary Figures 1A-C). Since we followed the same methodology with the Ossenkoppele R, et al. 2018, our ADNI-based evaluated threshold of 1.33 (1.3–1.36) for defining tau PET positivity was directly comparable and validated the threshold (1.34) proposed by Ossenkoppele R, et al. 2018 (difference within 0.7% from the published threshold).

**Supplementary Figures**

**Supplementary Figure 1**


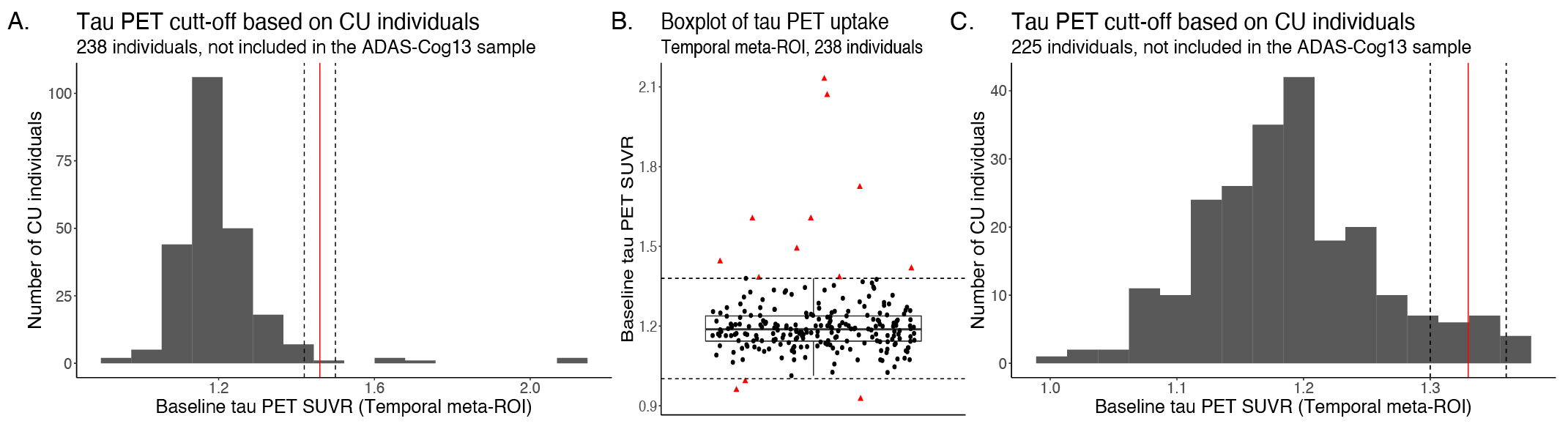


**Supplementary Figure 1**: Evaluation of an ADNI-based tau PET threshold using the mean + (2 × StD) in CU individuals approach for defining tau PET positivity. **A**. The distribution of tau PET SUVR values in the temporal meta-ROI of all CU individuals. **B**. Visualization and exclusion of outliers (red triangles) from the distribution plotted in A. The outliers were defined as tau PET SUVR values less than Q1 – (1.5 × IQR) or greater than Q3 + (1.5 × IQR). **C**. Evaluation of the tau PET threshold for defining tau PET positivity after the exclusion of outliers. The tau PET threshold (red line) was defined as 1.1882 + (2 × 0.0708) ≈ 1.33 (mean = 1.1882, StD = 0.0708). The gray zone was defined as 1.33 ± (2.5% × 1.33) ≈ (1.3–1.36) (dashed black lines). [ADNI = Alzheimer’s Disease Neuroimaging Initiative; SUVR = standard uptake value ratio, Q1 = quartile 1, Q3 = quartile 3, IQR = interquartile range, StD = standard deviation]

**Supplementary Figure 2**


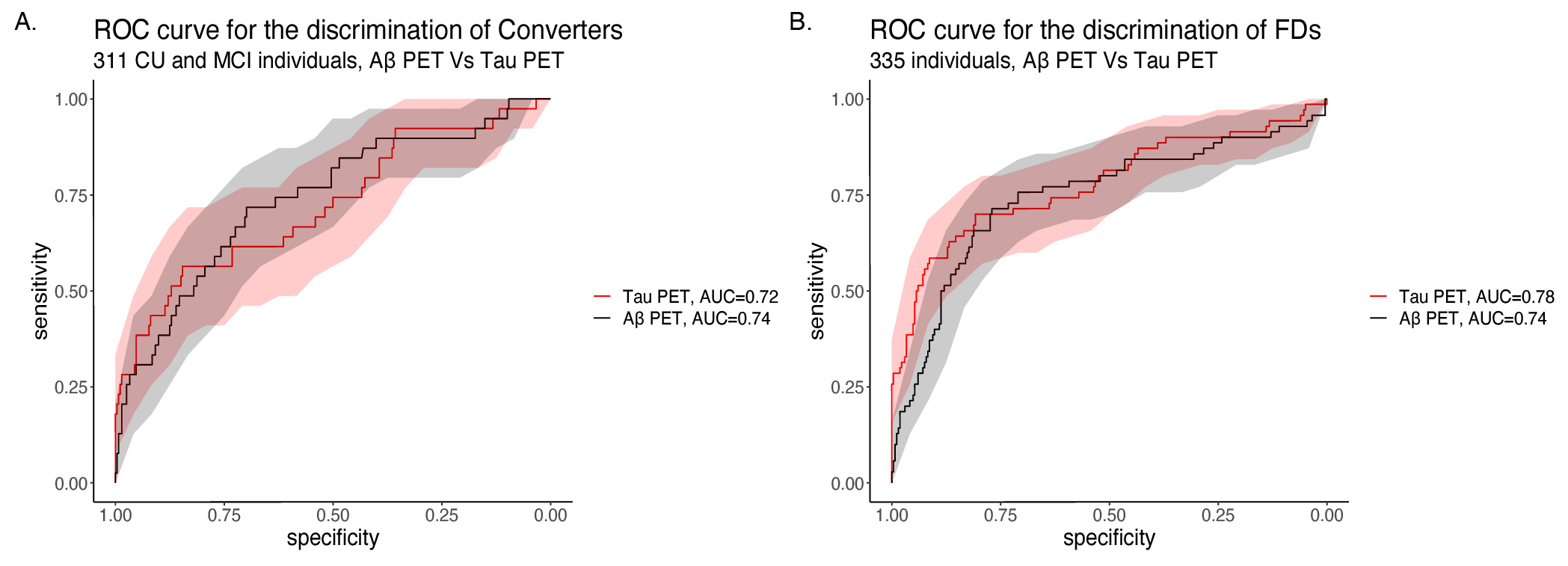


**Supplementary Figure 2**: Comparison of Αβ vs. tau PET for discriminating clinical conversion and fast cognitive decline using ROC analysis. Tau PET uptake was evaluated in the temporal meta-ROI in SUVR. Αβ PET uptake was measured in CL and represents the global Aβ burden. The ADAS-Cog13 score was used to evaluate the progress in cognitive decline (SD or FD). [Aβ = β-amyloid; ADAS-Cog13 = 13-item version of the Alzheimer’s Disease Assessment Scale-Cognitive Subscale; CL= centiloid; FD = fast decliner; SD = slow decliner; PET = positron emission tomography; ROI = region of interest; SUVR = standardized uptake value ratio; T = tau]

**Supplementary Figure 3**


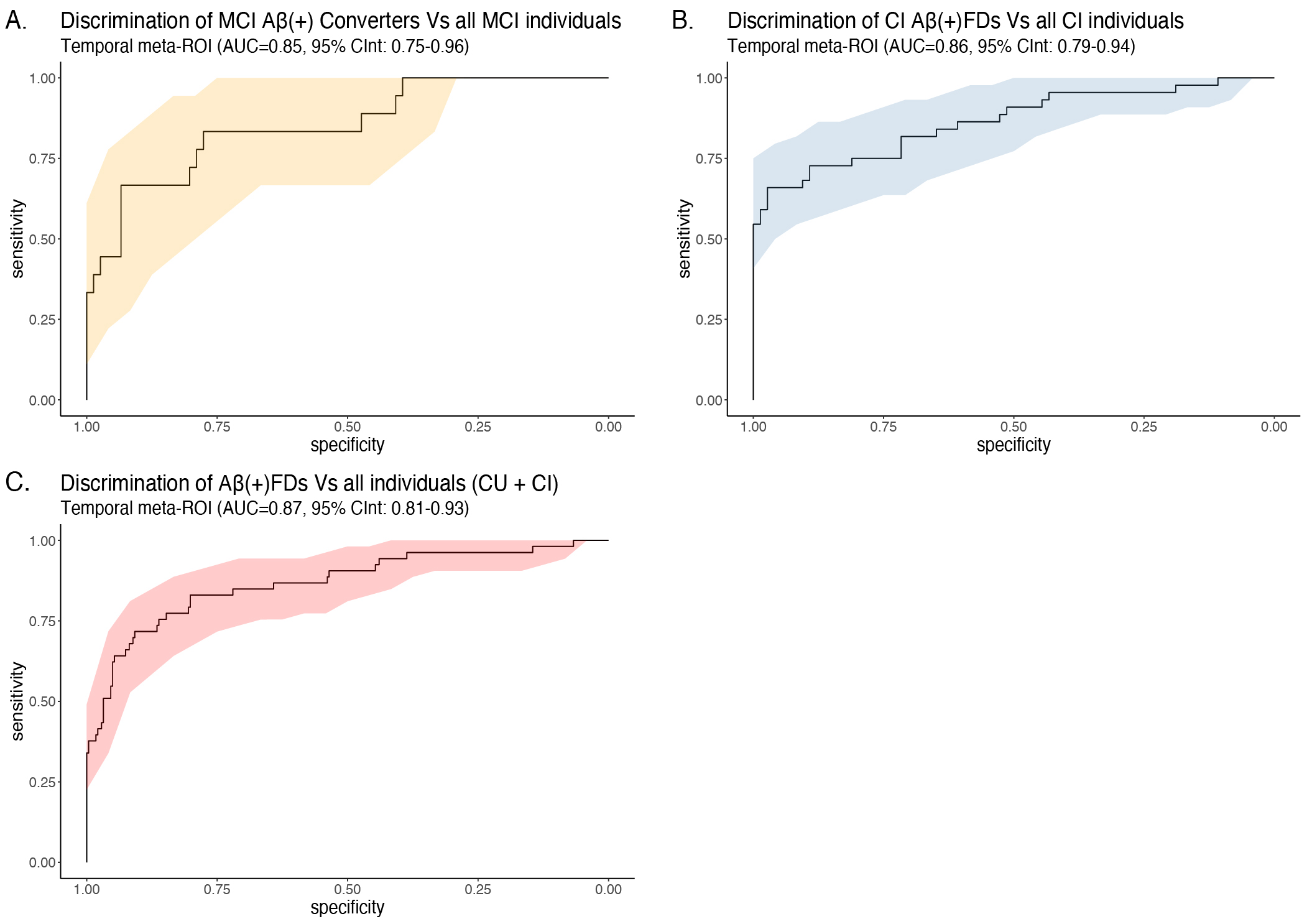


**Supplementary Figure 3**: ROC curves and AUC values with the corresponding confidence intervals (CInt) for the accuracy of discrimination (i.e., prognostic accuracy) of the baseline tau PET uptake in the temporal meta-ROI. Discrimination of A.) MCI Aβ(+) Converters among all MCI individuals, B.) CI Aβ(+)FDs among all CI individuals, and C.) Aβ(+)FDs among all individuals (CU + CI).

**Supplementary Figure 4**

**
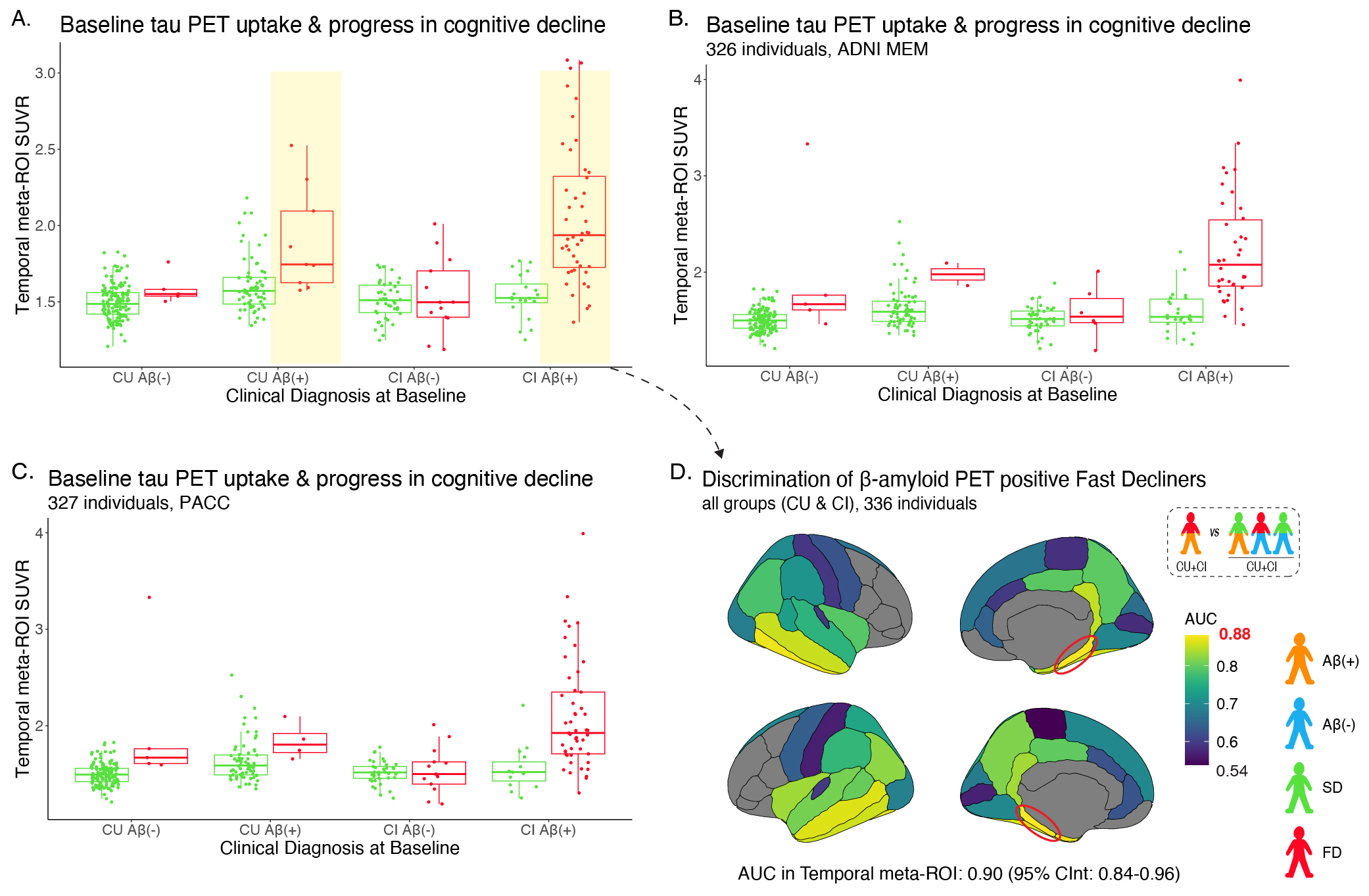
**

**Supplementary Figure 4**: Baseline tau PET uptake and progress in cognitive decline after applying correction for partial volume effects (PVC) to the tau PET data. The same pipeline as used in Figure 2 was used to cluster the individuals as FDs and SDs based on the **A**. ADAS-Cog13, **B**. ADNI MEM and **C**. PACC score. The Aβ(+)FDs, especially in the case of CI individuals, showed higher tau PET uptake in the temporal meta-ROI at baseline in relation to the other groups. **D**. The results of a ROC analysis illustrated in a brain atlas for the discrimination of Aβ(+)FDs among CU + CI individuals. The brain regions that are plotted in gray had no available tau PET data corrected for partial volume effect. For one of the individuals, only the tau PET PVC data were available and this is why the number of individuals is slightly different (n = + 1 in every cognitive score) in the PVC scenario. For the correction of partial volume effects, the Geometric Transfer Matrix (GTM) approach was used. [Aβ = β-amyloid; ADAS-Cog13 = 13-item version of the Alzheimer’s Disease Assessment Scale-Cognitive Subscale; ADNI MEM = Alzheimer’s Disease Neuroimaging Initiative episodic composite memory score; AUC = area under the curve; CInt = confidence interval; CI = cognitively impaired; CU = cognitively unimpaired; FD = fast decliner; PACC = Preclinical Alzheimer Cognitive Composite score; PET = positron emission tomography; ROC = receiver operating characteristic; ROI = region of interest; SD = slow decliner; SUVR = standardized uptake value ratio]

**Supplementary Figure 5**


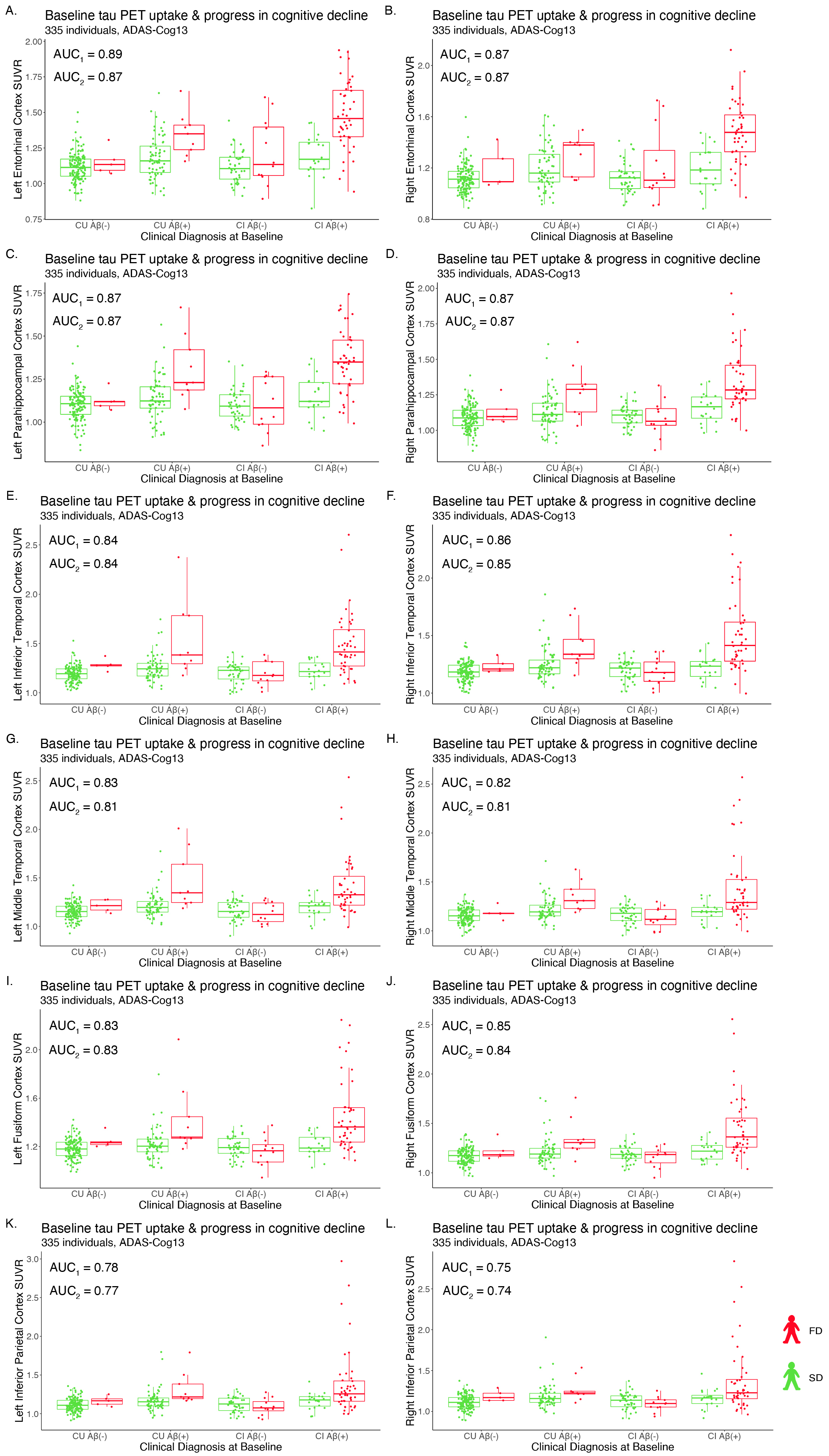


**Supplementary Figure 5**: Baseline tau PET uptake in ROIs composing the temporal meta-ROI and in other cortical brain areas and progress in cognitive decline. The Aβ(+)FDs present higher baseline tau PET uptake relative to the other groups [Aβ(-)FD/SDs and Aβ(+)SDs]. **A,B**. Left and right entorhinal cortex. **C,D**. Left and right parahippocampal cortex (parahippocampal cortex is not part of the temporal meta-ROI as the latter is defined in the ADNI protocol). **E,F**. Left and right inferior temporal cortex. **G,H**. Left and right middle temporal cortex. **K,L**. Left and right fusiform cortex. **I,J**. Left and right inferior parietal cortex. Cognitive decline profiling (SD/FD) is based on the ADAS-Cog13. AUC_1_ represents the accuracy of tau PET imaging for discriminating Aβ(+)FDs among all individuals (i.e., CU + CI). AUC_2_ represents the accuracy of tau PET imaging for discriminating Aβ(+)FDs among CI individuals. [ADNI = Alzheimer’s Disease Neuroimaging Initiative; AUC = area under the curve; Aβ = β-amyloid; CI = cognitively impaired; CU = cognitively unimpaired; FD = fast decliner; SD = slow decliner; ADAS-Cog13 = 13-item version of the Alzheimer’s Disease Assessment Scale-Cognitive Subscale; PET = positron emission tomography; ROI = region of interest; SUVR = standardized uptake value ratio]

**Supplementary Figure 6**


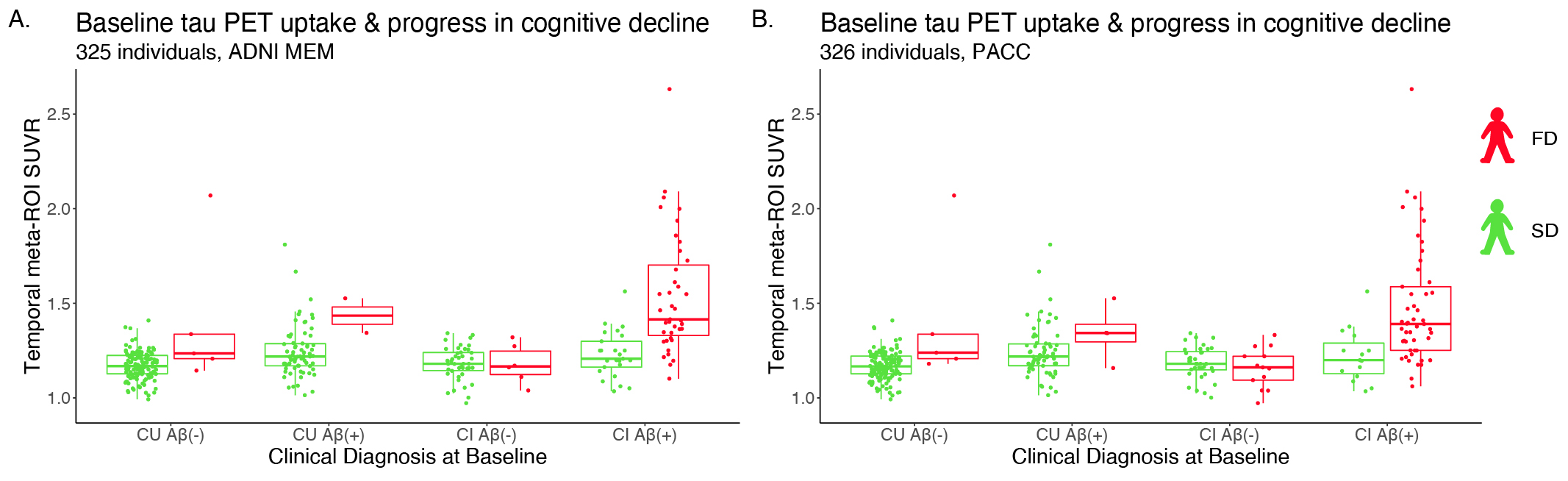


**Supplementary Figure 6**: Baseline tau PET uptake (without correction for partial volume effects) and progress in cognitive decline. The same pipeline as shown in Figure 2 was used to cluster the individuals into FD and SD based on the **A**. ADNI MEM and **B**. PACC score. The Aβ(+)FDs, especially those in the group of CI individuals, showed higher tau PET uptake in the temporal meta-ROI at baseline in relation to the other groups. [Aβ = β-amyloid; ADNI MEM = Alzheimer’s Disease Neuroimaging Initiative episodic composite memory score; CI = cognitively impaired; CU = cognitively unimpaired; FD = fast decliner; PACC = Preclinical Alzheimer Cognitive Composite score; PET = positron emission tomography; ROI = region of interest; SD = slow decliner; SUVR = standardized uptake value ratio]

**Supplementary Figure 7**


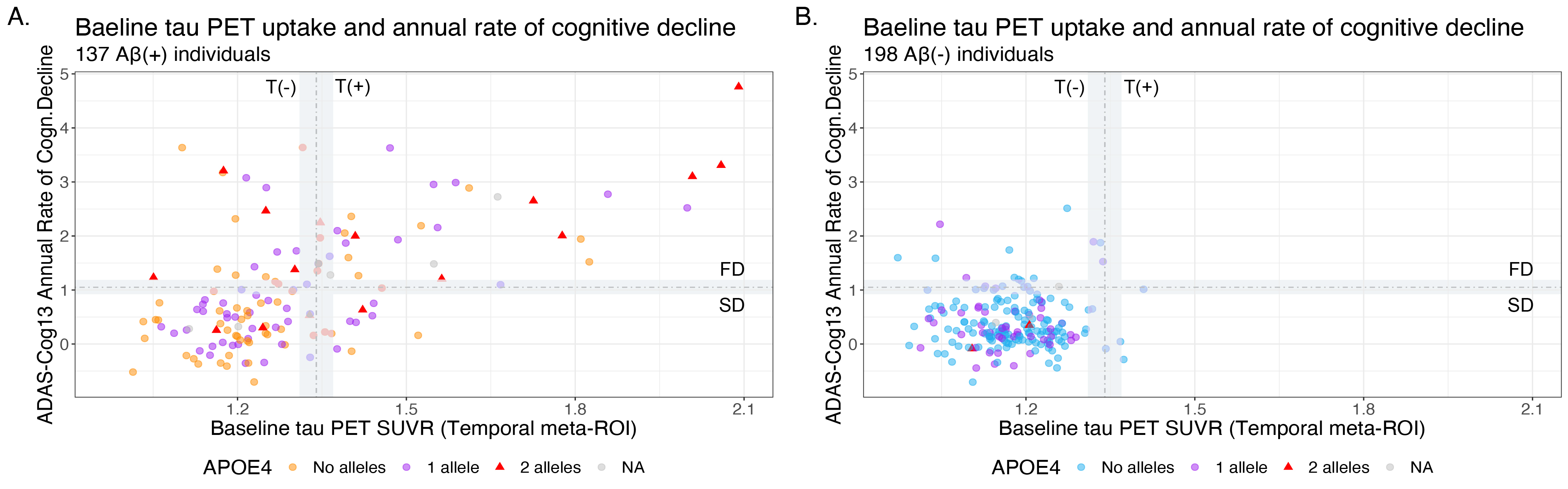


**Supplementary Figure 7**: The distribution of the APOE4 allele and the association between baseline tau PET uptake and annual rate of cognitive decline. The two graphs have the same axes scale so as to be comparable. **A**. Aβ(+) individuals and **B**. Aβ(-) individuals. (See also **Supplementary Table 4**.) [Aβ = β-amyloid; ADAS-Cog13 = 13-item version of the Alzheimer’s disease assessment scale-cognitive subscale; FD = fast decliner; PET = positron emission tomography; ROI = region of interest; SD = slow decliner; SUVR = standardized uptake value ratio; T = tau]

**Supplementary Figure 8**

**
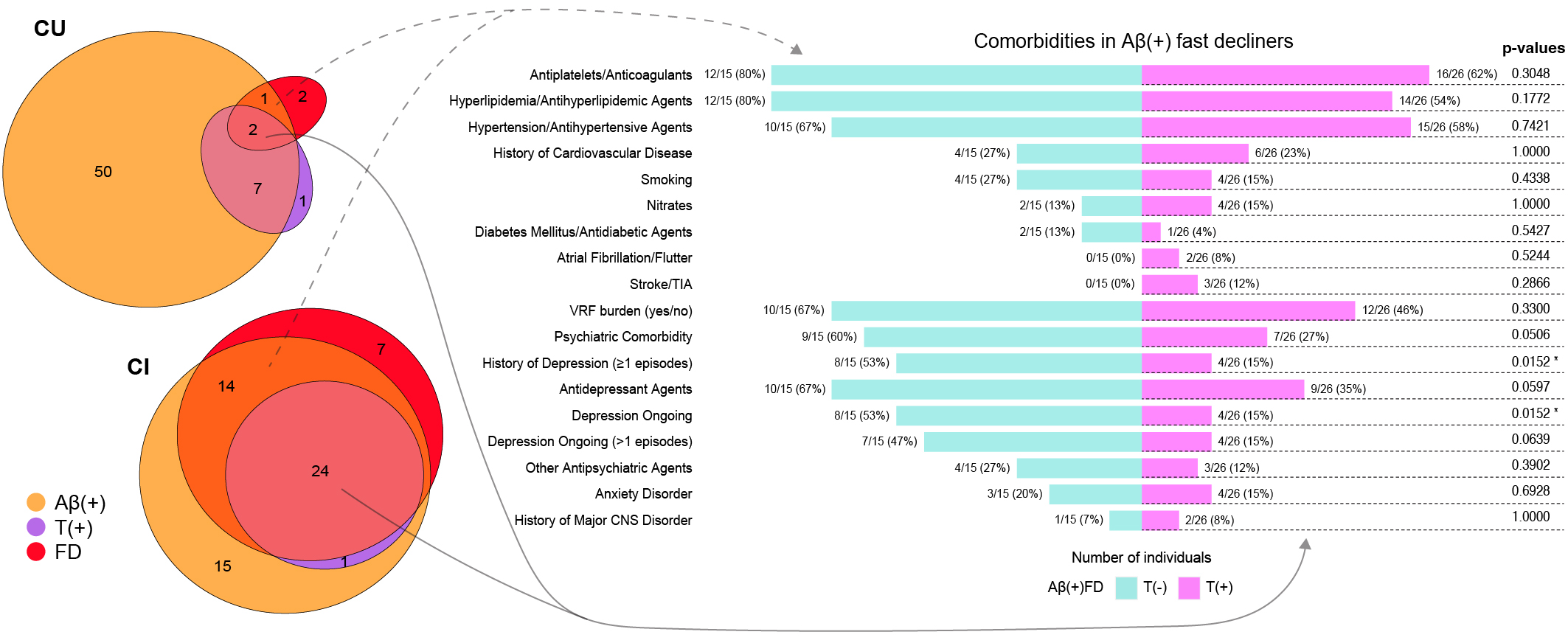
**

**Supplementary Figure 8**: A classification proposal: exploring the characteristics of Aβ(+)FDs. **Left (A)**: Venn-diagrams to illustrate the overlap of Aβ(+), T(+) and FDs in both CU and CI individuals. The size of each group is depicted proportional to the number of individuals it comprises. The individuals located in the gray zones of Figure 4E have been excluded. **Right (B)**: A comparison of comorbidities between Aβ(+)T(-)FDs and Aβ(+)T(+)FDs. The results of Fisher’s test for all categories are presented. Comorbidities were assessed for all individuals at the beginning of ADNI3; medications were assessed at enrollment and during the ADNI3 phase. The term psychiatric comorbidity includes individuals who had at least one psychiatric disorder; this includes all categories of depression and anxiety disorder and one individual [CU, Aβ(+)T(+)FD] who was diagnosed with seasonal affective disorder. [Aβ = β-amyloid; ADNI = Alzheimer’s Disease Neuroimaging Initiative; CI = cognitively impaired; CU = cognitively unimpaired; CNS = central nervous system; FD = fast decliner; T = tau; TIA = transient ischemic attack; VRF = vascular risk factor]

**Supplementary Figure 9**


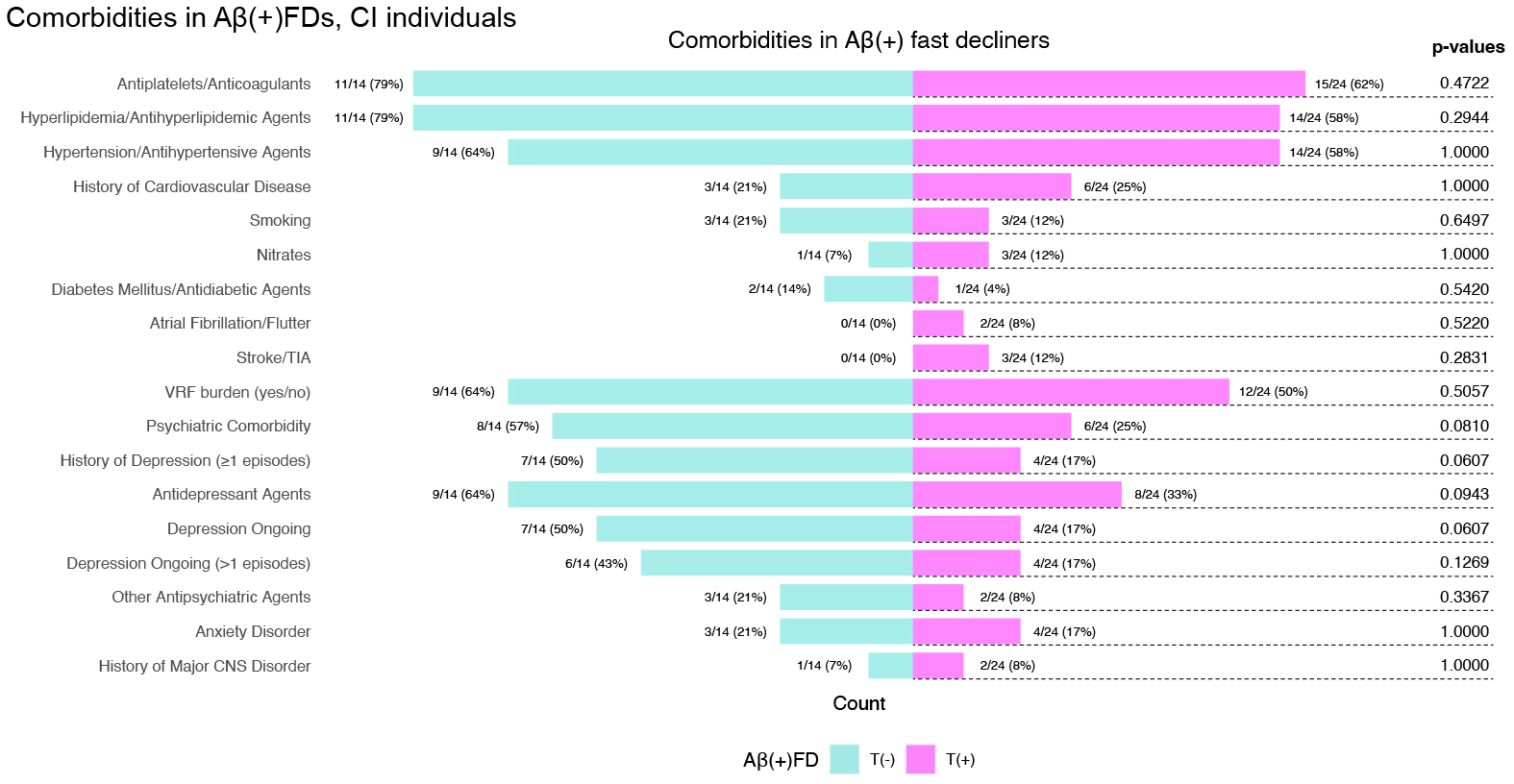


**Supplementary Figure 9**: A comparison of comorbidities between Aβ(+)T(-)FDs and Aβ(+)T(+)FDs in the group of CI individuals under study. The CI individuals located in the gray zones of Figure 4E have been excluded. The results of Fisher’s test for all categories are presented. Comorbidities were assessed for all individuals at the beginning of ADNI3; medications were assessed at enrollment and during the ADNI3 phase. The term psychiatric comorbidity includes individuals who had at least one psychiatric disorder; this includes all categories of depression and anxiety disorder. [Aβ = β-amyloid; ADNI = Alzheimer’s Disease Neuroimaging Initiative; CI = cognitively impaired; CNS = central nervous system; FD = fast decliner; T = tau; TIA = transient ischemic attack; VRF = vascular risk factor]

**Supplementary Tables**

**Supplementary Table 1**: ADNI files and R packages

| **Participants** |  |  |
| --- | --- | --- |
| **Category** | **What we used** | **Name of the ADNI file** |
| Demographics | Age, gender, education | PTDEMOG.21.01.22.csv |
| Clinical data | Clinical diagnosis | DXSUM_PDXCONV_ADNIALL.21.01.22.csv |
| Medical history | Medication | RECCMEDS.csv |
|  | Family history | FAMXHPAR.csv, FAMXHSIB.csv |
|  | ADNI3 initial assessment | INITHEALTH.csv |
| APOE genotype | Presence of APOE4 genes | APOERES.csv |
| Neuropsychological tests | ADAS-Cog13  (13-item version of the Alzheimer’s disease assessment scale-cognitive subscale) | ADAS_ADNIGO23_21.01.22.csv |
|  | ADNI MEM  (ADNI episodic composite memory score) | UWNPSYCHSUM_12_13_21.csv |
|  | PACC  (Preclinical Alzheimer Cognitive Composite) | ADNIMERGE package |
| **PET imaging biomarkers** |  |  |
| **PET** | **What we used** | **Name of the ADNI file** |
| β-amyloid PET | AV45 | UCBERKELEYAV45_01_14_21.csv |
|  | FBB | UCBERKELEYFBB_01_14_21.csv |
| tau PET | no PVC data  (no correction for partial volume effects) | UCBERKELEYAV1451_11_16_21.csv |
|  | PVC data  (corrected for partial volume effects) | UCBERKELEYAV1451_PVC_01_12_22.csv |
| **Software for analysis** |  |  |
| **R version** | **What we used** | **Name of the special R package** |
| R version 4.1.1 | Linear mixed effects models | lme4 |
|  | Gaussian mixture models | mixtools |
|  | Receiver operating characteristic analysis | pROC |
|  | Brain atlas | ggseg |
|  | Linear models | stats, emmeans |

**Supplementary Table 2**: Summary of the analysis design

| **Study analysis step-by-step** | | | |
| --- | --- | --- | --- |
| **Step** | **What** | **Why** | **How** |
| 1 | Evaluation of the annual rate of cognitive decline for all individuals | Defining a continuous measure for clustering into groups | Linear mixed effects models |
| 2 | Clustering individuals into clinically meaningfully groups (i.e., slow and fast decliners) | - Comparison of the baseline tau PET uptake between the different groups - Evaluation of the prognostic accuracy of a biomarker to discriminate between them - Extrapolation of outcomes at the individual level | Gaussian mixture models |
| 3 | Evaluation of the prognostic accuracy of a tau biomarker to discriminate a groups of high clinical relevance (i.e., MCI Aβ(+) Converters, Aβ(+)FDs) | Establishing a framework of clinical applicability for tau biomarkers | ROC |
| 4 | Defining threshold of tau positivity | - Validation of previously proposed thresholds - Establishing a framework of clinical applicability for tau biomarkers | Testing the applicability  of a previously published threshold in our population |
| 5 | Association of baseline tau PET uptake and annual rate of cognitive decline | Visualization of the impact of clustering | Linear models |
| 6 | Testing the overlap among the groups Aβ(+), T(+), and FDs | Establishing a framework of clinical applicability for tau biomarkers | Venn diagrams |
| 7 | Comparison of the prevalence of comorbidities and hypometabolism patterns suggestive of AD between Aβ(+)T(-)FDs and Aβ(+)T(+)FDs | Establishing a framework of clinical applicability for tau biomarkers | Table 2 |
| 8 | **Formulating a hypothesis**   - Tau positive individuals constitute a subgroup of fast decliners with evident Aβ pathology - Tau positivity can discriminate the individuals with AD-relevant cognitive decline (i.e., Aβ(+)T(+)FDs) - Non-AD comorbidities or the presence of mixed pathologies may contribute to or drive fast cognitive decline accompanied by isolated Aβ positivity - Further studies including multiple tau PET tracers and post-mortem data are needed to validate our hypothesis before the establishment of the clinical applicability of tau biomarkers | | |

**Supplementary Table 3**: Diagnostic accuracy of Aβ and tau PET imaging

|  | **Diagnostic Method (PET)** | **Population/Target** | **Value (95% CInt)** |
| --- | --- | --- | --- |
| **Sensitivity (Se)** | Aβ | FDs vs. SDs | 0.76 (0.64, 0.85) |
|  |  | Converters vs. Non-Converters | 0.72 (0.55, 0.85) |
|  | Tau | FDs vs. SDs | 0.49 (0.36, 0.61 ) |
|  |  | Converters vs. Non-Converters | 0.44 (0.28, 0.60) |
| **Specificity (Sp)** | Aβ | FDs vs. SDs | 0.68 (0.62, 0.74) |
|  |  | Converters vs. Non-Converters | 0.67 (0.61, 0.73) |
|  | Tau | FDs vs. SDs | 0.94 (0.91, 0.97) |
|  |  | Converters Vs Non-Converters | 0.92 (0.88, 0.95) |
| **Positive Predictive Value (PPV)** | Aβ | FDs vs. SDs | 0.39 (0.30, 0.47) |
|  |  | Converters vs. Non-Converters | 0.24 (0.17, 0.33) |
|  | Tau | FDs vs. SDs | 0.69 (0.55, 0.82) |
|  |  | Converters vs. Non-Converters | 0.44 (0.28, 0.60) |
| **Negative Predictive Value (NPV)** | Aβ | FDs vs. SDs | 0.91 (0.87, 0.95) |
|  |  | Converters vs. Non-Converters | 0.94 (0.90, 0.97) |
|  | Tau | FDs vs. SDs | 0.87 (0.83, 0.91) |
|  |  | Converters vs. Non-Converters | 0.92 (0.88, 0.95) |

Tau PET (18F-flortaucipir) positivity was defined as tau PET SUVR ≥ 1.34 in the temporal meta-ROI (Ossenkoppele et al. 2018). Aβ PET (18F-florbetapir or 18F-florbetaben) positivity was defined based on the ADNI cutpoints (i.e., 20 CL). The individuals located in the gray zones of Figure 4E have been included in the calculations of sensitivity, specificity, positive and negative predictive values concerning the fast or slow progress in cognitive decline.

**Supplementary Table 4**: The prevalence of the APOE4 allele in the groups of Supplementary Figure 7

| **Aβ PET** | **APOE4 alleles** | **Tau PET and progress in cognitive decline** | | | |
| --- | --- | --- | --- | --- | --- |
|  |  | ***T(-)*** | | ***T(+)*** | |
|  |  | ***SD*** | ***FD*** | ***SD*** | ***FD*** |
| ***Aβ(+)*** | *No allele* | 36 | 9 | 5 | 11 |
|  | *1 allele* | 32 | 6 | 5 | 12 |
|  | *2 alleles* | 3 | 4 | 1 | 8 |
| ***Aβ(-)*** | *No allele* | 129 | 11 | 4 | 0 |
|  | *1 allele* | 44 | 5 | 0 | 0 |
|  | *2 alleles* | 2 | 0 | 0 | 0 |

* Aβ = β-amyloid; FD = fast decliner; PET = positron emission tomography; SD = slow decliner; T = tau

**Supplementary Table 5**: Clinical characteristics of Aβ(-)T(-) fast decliners

| **Aβ/Tau PET status** | **Aβ(-)T(-) Fast decliners** *(N=17)*^1^ |
| --- | --- |
| **Baseline** |  |
| **Age, y** | 74.83 (8.04) |
| **Gender** |  |
| Male | 9 (53%) |
| Female | 8 (47%) |
| **Education, y** | 15.53 (3.3) |
| **Cognitive status** |  |
| CU | 5 (29%) |
| CI | 12 (71%) |
| **ADAS-Cog13** | 17.2 (6.97) |
| **APOE4 (≥1 allele)** | 5/16 (31%) |
| Not available | 1 |
| **Centiloids at Aβ PET** | -1.85 (13.46) |
| **Tau PET SUVR (temporal meta-ROI)** | 1.18 (0.1) |
| **FDG PET hypometabolism pattern** |  |
| Suggestive of AD | 4/13 (31%) |
| Not suggestive of AD | 9/13 (69%) |
| Not available | 4 |
| **Follow-up** |  |
| **Interval, y mon** | 35.4 (11.04) |
| **ADAS-Cog13 annual decline rate** | 1.49 (0.44) |
| **Medical history and medication use**^2^ |  |
| **Cerebrovascular disease risk factors** |  |
| Antiplatelets/Anticoagulants | 10 (59%) |
| Hyperlipidemia/Antihyperlipidemic agents | 13 (76%) |
| Hypertension/Antihypertensive agents | 10 (59%) |
| History of cardiovascular disease | 3 (18%) |
| Smoking | 1 (5.9%) |
| Nitrates | 1 (5.9%) |
| Diabetes mellitus/Antidiabetic agents | 5 (29%) |
| Atrial fibrillation/Flutter | 0 (0%) |
| Stroke/TIA | 0 (0%) |
| VRF burden (yes/no)^3^ | 9 (53%) |
| **Psychiatric comorbidities** |  |
| Psychiatric comorbidity | 7 (41%) |
| History of depression (≥1 episodes) | 6 (35%) |
| Antidepressant agents | 8 (47%) |
| Depression ongoing | 5 (29%) |
| Depression ongoing (>1 episodes) | 4/16 (25%) |
| Not available | 1 |
| GDS (short form) | 2.82 (1.81) |
| Other antipsychiatric agents | 3 (18%) |
| Anxiety disorder | 5 (29%) |
| **Other** |  |
| History of major CNS disorder | 1 (5.9%) |
| Medication for AD | 8 (47%) |

^1^ Mean (Standard deviation); n / N (%).The individuals located in the gray zones of Figure 4E have been included. ^2^ Comorbidities were assessed for all individuals at the beginning of ADNI3; medications were assessed at enrollment and during the ADNI3 phase. The term psychiatric comorbidity includes individuals who had at least one psychiatric disorder; this includes all categories of depression and anxiety disorder. ^3^ The presence of VRF burden was defined as the coexistence of two or more of the following conditions: i) cardiovascular disease, ii) hypertension (positive medical history or use of antihypertensive medication), iii) diabetes mellitus (positive medical history or use of antidiabetic medication), iv) hyperlipidemia (positive medical history or use of antihyperlipidemic medication), v) stroke or TIA, vi) smoking (ever or never), vii) atrial fibrillation, and viii) left ventricular hypertrophy. [Aβ = β-amyloid; ADNI = Alzheimer’s Disease Neuroimaging Initiative; CI = cognitively impaired; CU = cognitively unimpaired; CNS = central nervous system; FD = fast decliner; GDS = geriatric depression scale; T = tau; TIA = transient ischemic attack; VRF = vascular risk factors]

**Supplementary Table 6**: Follow-up tau PET scans in Aβ(+)T(-)FDs (n=19) and Aβ(-)T(-)FDs (n=17)

| **Baseline tau PET scan** | **Follow-up tau PET scans** | | | | | | |
| --- | --- | --- | --- | --- | --- | --- | --- |
|  | **1-year** | | **2-years** | | **3 years** | | **4 years** |
|  | **Aβ(+)T(-)FDs (9/19 individuals)** | | | | | | |
| **4 years follow-up** | 2 individuals | | | | | | |
| Negative | - | - | | - | | Negative | |
| Negative | Negative | Negative | | - | | Negative | |
| **3 years follow-up** | 1 individual | | | | | | |
| Negative | Negative | - | | Negative | | - | |
| **2 years follow-up** | 5 individuals | | | | | | |
| Negative | Negative | | Negative | | - | | - |
| Negative | Negative | | Negative | | - | | - |
| Negative | Negative | | Negative | | - | | - |
| Negative | Negative | | Negative | | - | | - |
| Negative | - | | Negative | | - | | - |
| **1 year follow-up** | 1 individual | | | | | | |
| Negative | Negative | | - | | - | | - |
|  | **Aβ(-)T(-)FDs (6/17 individuals)** | | | | | | |
| **4 years follow-up** | 1 individual | | | | | | |
| Negative | - | | - | | - | | Negative |
| **2 years follow-up** | 3 individuals | | | | | | |
| Negative | - | | Negative | | - | | - |
| Negative | Negative | | Negative | | - | | - |
| Negative | Negative | | Negative | | - | | - |
| **1 year follow-up** | 2 individuals | | | | | | |
| Negative | Negative | | - | | - | | - |
| Negative | Negative | | - | | - | | - |

Tau PET positivity was defined as tau PET SUVR ≥ 1.34 in the temporal meta-ROI (Ossenkoppele et al. 2018 ).

Aβ = β-amyloid; T= tau

**Supplementary Table 7**: Baseline cognition Aβ(+)T(+)FDs vs. Aβ(+)T(+)SDs

|  | **Aβ(+)T(+)FDs** (n = 34)^1^ | **Aβ(+)T(+)SDs** (n = 11)^1^ |
| --- | --- | --- |
| **Cognitive status at baseline** | | |
| CU | 5/34 (15%) | 9/11 (82%) |
| CI | 29/34 (85%) | 2/11 (18%) |
| **Baseline ADAS-Cog13 score** | 23.33 (16.75–26.83) | 8.33 (5.84–9.84) |

^1^n/N (%); Median (IQR).

Aβ = β-amyloid; ADAS-Cog13 = 13-item version of the Alzheimer’s disease assessment scale-cognitive subscale; CI = cognitively impaired; CU = cognitively unimpaired; IQR=interquartile range; T= tau
